# Supplementary material for: Creation of an unexpected plane of enhanced covalency in cerium(III) and berkelium(III) terpyridyl complexes
Source: Nat Commun. 2021 Dec 10;12:7230. doi: 10.1038/s41467-021-27576-y (PMC8664847; doi:10.1038/s41467-021-27576-y)
Supplement: Supplementary file 1 — Supplementary Information [file 41467_2021_27576_MOESM1_ESM.pdf]

Creation of an Unexpected Plane of Enhanced Covalency in Cerium(III) and Berkelium(III) Terpyridyl Complexes

Alyssa N. Gaiser,[1] Cristian Celis-Barros,[1] Frankie D. White,[1] Maria J. Beltran-Leiva,[1] Joseph M. Sperling,[1] Sahan R. Salpage,[1] Todd N. Poe,[1] Daniela Gomez-Martinez,[1] Tian Jian,[2] Nikki J. Wolford,[3] Nathaniel J. Jones,[1] Amanda J. Ritz,[1] Robert A. Lazenby,[1] John K. Gibson,[2] Ryan E. Baumbach,[4] Dayán Páez-Hernández,[5] Michael L. Neidig,[3] Thomas E. Albrecht-Schönzart[1,*]

AFFILIATIONS

[1] Department of Chemistry and Biochemistry, Florida State University, Tallahassee, Florida 32306, USA, [2] Chemical Sciences Division, Lawrence Berkeley National Laboratory, Berkeley, California 94720, USA, [3] Department of Chemistry, University of Rochester, Rochester, NY, 14627, USA, [4] National High Magnetic Field Laboratory, Tallahassee, Florida 32310, USA, [5] Center for Applied Nanosciences, Universidad Andres Bello, Santiago, República 275, Chile

SUPPLEMENTARY INFORMATION

Contents

1. ADDITIONAL DISCUSSION 3
- 1.1 Theory 3
  - 1.1.1 Electronic structure 3
  - 1.1.2 Magnetism of Ce1 4
  - 1.1.3 QTAIM. 5
- 1.2 Experimental 6
  - 1.2.1 Collision-Induced Dissociation (CID) Mass Spectrometry 6
  - 1.2.2 Magnetic Circular Dichroism 9
  - 1.2.3 Electrochemistry 9
  - 1.2.4 Infrared Spectroscopy. 10

2. SUPPLEMENTARY METHODS 10

2.1 Experimental 10
2.1.1 Synthesis and Crystal Structure 10
2.1.2 Magnetism 13
2.1.3 Collision-Induced Dissociation Mass Spectrometry-Gas Phase Experimental Method 13
2.1.4 Magnetic Circular Dichroism..14
2.1.5 Electrochemistry..14
2.1.6 EPR Spectroscopy 15
2.1.7 Solid State UV-visible-NIR Spectroscopy 15
2.1.8 Powder X-ray Diffraction 16
2.1.9 ATR-IR Measurements...16
2.2 Computational Details...16
2.2.1. Wavefunctions. 16
2.2.2 Molecular Properties. 17
3. SUPPLEMENTARY FIGURES..19
Supplementary Fig. 1 | CASSCF natural orbitals of Bk1. 19
Supplementary Fig. 2 | CASSCF natural orbitals of Ce1..20
Supplementary Fig. 3 | Selected spin-orbit natural orbitals (SONOs) for Ce1. 21
Supplementary Fig. 4 | Selected spin-orbit natural orbitals (SONOs) for Bk1.........................22
Supplementary Fig. 5 | Calculated magnetic susceptibility (χT and χ^{-1}) for Ce1. 23
Supplementary Fig. 6 | Calculated magnetic susceptibility (χT and χ-1) for Bk1. 24
Supplementary Fig. 7 | Temperature dependent DC magnetic susceptibility χ = M / H. 25
Supplementary Fig. 9 | CID mass spectra of $[M(terpy^*)_2(NO_3)_2]^+$ 28
Supplementary Fig. 10 | CID mass spectra of $[M(terpy^*)_2(NO_3)_2]^+$ 29
Supplementary Fig. 11 | CID mass spectra of [M(terpy)(NO3)2]+ for (a) M = Ce and (b) M = Eu, both at a nominal CID voltage of 0.35 V. 30
Supplementary Fig. 12 | CID mass spectra of $[M(terpy^*)(NO_3)_2]^+$ for (a) M = Ce and (b) M = Eu, both at a nominal CID voltage of 0.35 V. 31
Supplementary Fig. 13 | CID mass spectra of $[M(terpy)(NO_3)_2]^+$ 32
Supplementary Fig. 14 | CID mass spectra of $[M(terpy^*)(NO_3)_2]^+$...33
Supplementary Fig. 15 | CID mass spectra of (a) $[H_{terpy}]^+$ at a nominal CID voltage of 0.95 V, and (b) $[H_{terpy^*}]^+$ at 0.65 V. 34
Supplementary Fig. 16 | UV-vis Magnetic Circular Dichroism spectra of Ce1. The UV-vis MCD spectrum of a fluorolube mull of Ce1. 35
Supplementary Fig. 17 | Ce2 Structure and plane. 36
Supplementary Fig. 18 | Cyclic voltammograms of 5 mM Ce1 complex................................37

Supplementary Fig. 19 | Cyclic voltammograms of 0.5 mM and 5 mM Ce1 complex. 38
Supplementary Fig. 20 | Experimental and calculated Powder X-ray Diffraction (PXRD) patterns of Ce1. 39
Supplementary Fig. 21 | ATR-IR Spectra of Ce1 (top) and Ce2 (bottom) from 2,400 – 3,500 cm^{-1}. 40
Supplementary Fig. 22 | ATR-IR Spectra of Ce1 (top) and Ce2 (bottom) from 500 – 1,500 cm^{-1}. 41
4. SUPPLEMENTARY TABLES 42
Supplementary Table 1 | Spin-orbit states for Bk1. 42
Supplementary Table 2 | Molecular electronic states derived from the $^7F_6$ in Bk1. 43
Supplementary Table 3 | Composition of the three Kramer's doublets of Ce1. 44
Supplementary Table 4 | Ab-initio calculated crystal-field parameters B(k,q), where *k* corresponds to the rank and *q* the projection of the operator. 45
Supplementary Table 6 | QTAIM metrics at the bond critical point (BCP) for Ce1. 47
Supplementary Table 7 | QTAIM metrics at the bond critical point (BCP) for Bk1. 48
Supplementary Table 8 | Interacting Quantum Atom (IQA) energy decomposition analysis. ..49
5. SUPPLEMENTARY REFERENCES 56

1. ADDITIONAL DISCUSSION

1.1 Theory

1.1.1 Electronic structure. The electronic structure calculations of heavy actinide complexes are a current topic that requires the use of methodologies that rigorously consider electronic correlation and relativistic effects. In general, studies in this area are scarce and the earliest references date from the 1980s when the free ion approximation was the most popular approach to describe the electronic structure of lanthanide and actinide compounds.[1] However, in actinides compounds the application of these approximations may introduce problems in the correct assignment of the electronic states and spectroscopic properties due to the possibility of important metal ligand interaction. The electronic structures of **Ce1** and **Bk1** were examined using relativistic *ab-initio* wavefunction calculations (CASSCF) including spin-orbit (SO) coupling and dynamic correlation (PT2).

According to Hund's rule and in a scalar relativistic framework the ground state of Bk(III) free ion is ^7F (L = 3, S = 3) and is followed by a ^5D multiplets ~17300 cm^{-1} higher in energy. The inclusion of spin-orbit coupling, which is crucial to describe the electronic structure of heavy elements (ξ

~3300 cm^{-1} in Bk (III) ion), lifts the degeneracy of the spin-free states. This yields a series of multiplets characterized by the total angular momentum (J). The ground multiplet for Bk(III) ($^7F_6$) is followed by the $^5D_4$ (~18500 cm^{-1}), $^5D_3$ (~24700 cm^{-1}), and $^3L_{10}$ (~25500 cm^{-1}). The free-ion calculation agrees with a systematic study performed by Suchotcki et al. predicting the energy levels schemes of lanthanide and actinides with spectroscopic quality at CASSCF (CAS-PT2) level of theory (Supplementary Table 1).[2]

When the ligand field is considered in the model the 2J+1 degeneracy of the free ion multiplets is completely lifted and several low-lying states are stabilized, which makes the analysis of the spectroscopic experimental data more complex. Even when for many purposes the free-ion model can be extended to analyze the molecular properties, this approach is more appropriate when the metal-ligand orbital interaction is predominantly ionic. Since recent evidence shows that heavy actinides such as berkelium interact more covalently with ligands certain soft-donor ligands, the molecular view is preferable due to the bonding interaction involving the 5*f* orbitals with the ligands.[3–6]

Molecular calculations were performed considering two active spaces, the minimal consisting of eight electrons in seven 5*f* orbitals and the expanded active space that includes the 6*d* shell to evaluate the role of these orbitals in the electronic structure of the complex. It is interesting that only including the 6*d* shell in the active space enable us to see the non-Kramers doublet nature (accidental degeneracy) of the three six first states (Supplementary Table 2). The dimension of the active space as well as the nature of the excited states influence the total splitting of the $^7F_6$ free-ion multiplet. Furthermore, the importance of the spin-orbit coupling to the molecular states is observed in the 9% contribution coming from the quintuplet states derived from the $^5G_6$ free-ion multiplet. Because of the doublet character of the ground, first and second excited states a pseudo-spin ½ approximation can be used to analyze the magnetic properties (See main text).

Even though the SO-CAS(8,12) wavefunction makes possible the assessment of the $5f^8 \longrightarrow 5f^7 6d^1$ electronic transitions (45000 – 50000 cm^{-1}), the assignment is not expected to be accurate due to the active space lacking important orbital interactions between metal-6*d* and terpy*-π orbitals. It is expected that the inclusion of the orbital ligands in a balanced way should introduce changes in the position of these electronic transition bands.

1.1.2 Magnetism of Ce1. Based on the electronic structure of **Ce1**, the substates corresponding to $^2F_{7/2}$ lie at ~ 2200-3000 cm^{-1}, which means that the thermal population of these states has no influence on the magnetic properties of the complex. The wavefunction analysis in

terms of the |J, M_J> components show a strong mixing between terms that is responsible for the non-axial magnetic response of the complex (Supplementary Table 3).

The crystal-field parameters calculated for **Ce1** explain the mixed character of the states. The axial and nonaxial crystal field parameters are of the same magnitude and the rank 2 parameters are the largest (Supplementary Table 4), that explain the small anisotropic character of the ground Kramers doublet and the strong mixing between the |J, M_J> terms in the three low lying states showed in Supplementary Table 3.

The calculated magnetic susceptibility for **Ce1** shows a typical behavior for a paramagnetic system and is in good agreement with the experimental determination (at least below 200 K). SOC-CAS and SOC-PT2 methods predict similar results, and the expansion of the active space (including 5*d* orbitals) has no significant impact on the χ^{-1} versus T plot (Supplementary Fig. 5). Below ~100 K the magnetic moment (χT) rapidly decreases, which can be attributed to the thermal depopulation of the low-lying excited states, whereas at higher temperatures the susceptibility converges to a linear behavior. Therefore, the expected proportional relation of χ^{-1} vs T is observed (Supplementary Fig. 5). The calculated magnetic moment is 2.513 μ_B that is in agreement with the experimental value 2.2 μ_B, while the χT value in the limit low temperature corresponds to ~ 0.313 cm^3 K mol^{-1}, which agrees with the expected value for a doublet state which represent a pseudospin 1/2 (0.38 cm^3Kmol^{-1}).

The analysis of the natural orbital (NO) occupations shows a more homogeneous electron distribution of the 5*f* shell that results in a more spherical 5*f* electron density. However, the orbitals with maximal distribution in the plane composed by terpyridine and water molecule show larger occupation numbers, which introduces some asphericity in the electron distribution with a slight oblate shape (Supplementary Fig 3).

1.1.3 QTAIM. To better understand how covalency can be related to the atoms-in-molecules theory, we briefly highlight some important aspects of it. Firstly, one of the important metrics that is useful to understand covalency is the delocalization index, δ(r), that is analog to the bond order descriptor. The electron density at the bond critical point (BCP) is contrasted to this value to understand whether an increase/decrease in the bond order is accompanied by an accumulation/depletion of the electron density in the bond. This is important to compare the strength of the bonds.

Due to the intrinsic difference between lanthanides and actinides in terms of their corresponding *f*-orbital radial extents, it is expected for actinides to have these electrons more readily interacting

with the local environment. Furthermore, the fact that the 5*p*/6*p* shells are semi-core in nature, i.e. their radial densities extend beyond the corresponding 4*f* /5*f* shells but lie deep down in terms of energy with respect to valence region, affects the exposure of the *f* electrons making their engagement in bonding more secondary. This results in the formation of partial covalent bonds rather than the formal ones observed in, for example, some transition metal complexes.[7] Within the QTAIM formalism, this partial covalent character is seen in the ratio between potential (V) and kinetic (G) energy densities, while a value of 1 represents the transition from an ionic to a partially covalent bond; a value of 2 represents the transition between a partial to a formally covalent bond. Negative values for the total energy density, H(r) = V(r) + G(r), also provide an estimation of the covalent interaction that can be seen as a measure of the stabilization of the electron density at the BCP.

1.2 Experimental

1.2.1 Collision-Induced Dissociation (CID) Mass Spectrometry

Gas Phase Experimental Results. Gas-phase cation complexes $[M(L)_n(NO_3)_2]^+$ (M=Ce or Eu; L=2,2':6'2''-terpyridine (terpy) or 4'-(4-nitrophenyl)-2,2':6'2''-terpyridine (terpy*); n=1 or 2) were produced by ESI and subjected to CID in a quadrupole ion trap mass spectrometer. CID fragmentation of metal-ligand coordination complexes can elucidate binding between metal and ligand. Specifically, stronger metal-ligand binding results in reduced susceptibility to fragmentation and a lower yield of the ligand-elimination CID product. CID results for $[M(L)_2(NO_3)_2]^+$, shown in Supplementary Fig. 8-10, can be summarized by primary unimolecular dissociation reaction (1) and secondary fragmentation reaction (2), where L = terpy or terpy*.

$$[M^{III}(L)_2(NO_3)_2]^+ \rightarrow [M^{III}(L)(NO_3)_2]^+ + L \quad (1)$$

$$[M^{III}(L)(NO_3)_2]+ \rightarrow [M^{IV}O(L)(NO_3)]^+ + NO_2 \quad (2)$$

From Supplementary Fig. 8 it is apparent that CID of $[M(terpy)_2(NO_3)_2]^+$ via reaction (1) occurs for both M=Ce and Eu. Secondary CID reaction (2) is observed for M=Ce but not for M=Eu. As reported previously, [8] elimination of NO_2 from cerium(III) nitrate corresponds to formation of a Ce(IV) oxo complex, here $[Ce^{IV}O(terpy)(NO_3)]^+$. The efficiency of reaction (2) reflects the ease of oxidation from M(III) to M(IV) as indicated by the M(IV/III) standard

reduction potential, which is much lower for Ce (1.7 V) versus Eu (estimated as ca. 6.4 V). The relative efficiency of reaction (1) under the same CID conditions is indicated by the product yield, which is defined here as the intensity of the CID product relative to the total ion intensity of product and undissociated complex. When secondary CID reaction (2) is observed, the product yield for primary reaction (1) is the sum of those for (1) and (2). The resulting yields for reaction (1) (L = terpy) are ~30% for M=Ce and ~47% for M=Eu. The higher CID efficiency for the Eu complex indicates lower binding energy of terpy in $[Eu(terpy)_2(NO_3)_2]^+$ versus $[Ce(terpy)_2(NO_3)_2]^+$.

CID results for the $[M(terpy^*)_2(NO_3)_2]^+$ were obtained under the same conditions as discussed above for the terpy complexes. The results, shown in Supplementary Fig. 9, reveal complete dissociation according to reaction (1). This higher CID efficiency for L = terpy* versus terpy indicates a lower binding affinity of both Ce and Eu for terpy* versus terpy in the gas-phase $[M(L)_2(NO_3)_2]^+$ complexes. It should be emphasized that such intrinsic binding affinity in isolated gas-phase complexes may be substantially perturbed by environmental effects in condensed phases, possibly to the extent that relative ligand binding energies can be inverted in condensed phase. As for terpy complexes, also for L = terpy* metal oxidation reaction (2) occurs for Ce but not Eu. In view of complete dissociation seen in Supplementary Fig. 9, results were obtained using a lower CID voltage of 0.25 V as shown in Supplementary Fig. 10, where the yield for reaction (1) is ~20% for the Ce complex and ~33% for the Eu complex. As with terpy, the CID results indicate a higher binding energy for Ce versus Eu with terpy*.

Supplementary Fig. 11-14 show CID results for complexes containing the same metals and ligands as above, but with composition $[M(L)(NO_3)_2]^+$, which are also the products of ligand elimination reaction (1). Using the same conditions as in Supplementary Fig. 8-9 there is no detectable ligand elimination via hypothetical reaction (3), which indicates stronger ligand binding in $[M(L)(NO_3)_2]^+$ versus $[M(L)_2(NO_3)_2]^+$. Reaction (2), which is now a primary CID process, occurs with both terpy and terpy* for M=Ce, but not for M=Eu. Also apparent in Supplementary Fig. 11 is minor loss of a second NO_2 to yield $[CeO_2(terpy)]^+$; the intriguing CeO_2^+ moiety that is presumably the core of this species has been considered previously. [9] As reaction (3) was not observed under the same conditions as reaction (2) the results in Supplementary Figs. 13-14 were acquired at a significantly higher CID voltage of 0.95 V. Even at this much higher CID energy there is still no evidence for reaction (3). The results indicate significantly weaker binding of ligands L in

$[M^{III}(L)_2(NO_3)_2]^+$ versus $[M^{III}(L)(NO_3)_2]^+$. Such decreasing binding energy with increasing number of ligands and charge donation to the metal center is typical. For example, the energy for addition of a water molecule is -134 kJ/mol to bare UO_2^+, but only -48 kJ/mol to tetrahydrated $[(UO_2)(H_2O)_4]^+$.[10] Apparent in Supplementary Figures 13-14 is elimination of NO_3 from $[Eu^{III}(L)(NO_3)_2]^+$ to yield $[Eu^{II}(L)(NO_3)]^+$ via reaction (4), which is not observed for the Ce complexes. The occurrence of reaction (4) reflects the standard reduction potentials, -0.34 V for Eu(III/II) and ca. -3.2 V for Ce(III/II). Other CID processes for $[Eu(L)(NO_3)_2]^+$ apparent in Supplementary Fig. 13-14 are due to ligand fragmentations.

$$[M^{III}(L)(NO_3)_2]^+ \rightarrow [M^{III}(NO_3)_2]^+ + L \qquad (3)$$

$$[M^{III}(L)(NO_3)_2]^+ \rightarrow [M^{II}L(NO_3)]^+ + NO_3 \qquad (4)$$

A particularly notable result apparent in Supplementary Fig. 13-14 is the very different redox chemistry observed for the Ce(III) and Eu(III) complexes with the same compositions and under the same CID conditions. Specifically, reaction (2) is observed for M=Ce while reaction (4) occurs for M=Eu. In the same chemical environment and under the same circumstances, oxidation from Ce(III) to Ce(IV) is manifested, while reduction of Eu(III) to Eu(II) occurs. Disparities in redox chemistry of the constituent metals are clearly expressed in the CID decomposition pathways for these nitrate complexes.

Supplementary Fig. 15 shows CID results of protonated ligand $[Hterpy]^+$ and $[Hterpy^*]^+$. The CID results for $[Hterpy^*]^+$ are comparable to those for $[Eu(terpy^*)(NO_3)_2]^+$. However, NO_3 loss does not occur for the bare protonated ligand, in accord with the attribution of this channel to metal reduction reaction (4). In contrast, CID of $[Hterpy]^+$ and $[Eu(terpy)(NO_3)_2]^+$ are rather disparate, suggesting rather different fragmentations for a terpy ligand when alternatively bound to a proton or a metal cation. As CID of the Ce complexes with one terpy or terpy* is dominated by nitrate anion ligand decomposition rather than neutral terpy or terpy* ligand decomposition, the CID results for the Ce complexes are necessarily not comparable to CID of bare protonated ligands.

A summary interpretation of the key CID results is the following: (1) Stronger metal-ligand binding is found in $[M(L)_2(NO_3)_2]^+$ for M = Ce versus M = Eu with both ligands, L = terpy and L = terpy*; (2) Stronger metal-ligand binding is found in $[M(L)_2(NO_3)_2]$+ for L = terpy versus L= terpy* with both M = Ce and M = Eu; (3) Stronger metal-ligand binding is found

for both M = Ce and M = Eu, and both L = terpy and L = terpy* in mono-ligand complexes $[M(L)(NO_3)_2]^+$ versus di-ligand complexes $[M(L)_2(NO_3)_2]^+$; and (4) Characteristic oxidation states Ce(IV) and Eu(II) are revealed by elimination from nitrate complexes of NO_2 and NO_3, respectively. It should be emphasized that the gas-phase results are for isolated species and do not consider solvent or lattice effects that are important in condensed phases. Furthermore, compositions and charge states of gas-phase and condensed phase complexes may be different. Although condensed phase chemistry may be quite different due to environmental perturbations, chemistry of isolated gas-phase complexes reveals intrinsic properties such as the strength of ligand binding to a metal center absent secondary environmental interactions.

1.2.2 Magnetic Circular Dichroism

The MCD spectrum can be fit to six transitions as predicted by the computational analysis. Assignments with respect to the calculated transitions can be seen in Supplementary Table 5. It is important to note that the tail at low energy is due to scattering.

All of the assigned transitions are ligand to metal apart from the transitions at 27500 cm^{-1} and 33450 cm^{-1}, which are metal to ligand transitions.

Additionally, theory predicts a transition at 40518.6 cm^{-1} that is not observed in the experimental MCD spectrum most likely because it is outside of the experimental range.

1.2.3 Electrochemistry.

While data presented for both Bk1 and Ce1 complexes in the main text used solutions containing 100 mM supporting electrolyte concentration, a higher concentration of 500 mM was also studied for Ce1. A high supporting electrolyte concentration is important for decreasing solution resistance and eliminating migration of the redox active species. Solvents with a low dielectric constant require higher supporting electrolyte concentrations to compensate for this. Cyclic voltammograms are shown in Supplementary Fig. 18 for the Ce1 complex in both 100 mM and 500 mM TBA PF_6. It was observed that the peak-to-peak separation increases slightly, although the behavior under both concentrations showed far from ideal reversibility, validating the result in the main text.

Supplementary Fig. 19 shows CVs for 0.5 mM (blue) and 5 mM Ce1 complex (red). The anodic and cathodic peak magnitudes increase as the concentration increases, indicating that peak observed is the complex, rather than the electrolyte solution. The addition of

higher concentration of complex also showed that more water was present in the solution, given the increased current magnitudes at the limits of the measurement window.

1.2.4 Infrared Spectroscopy.

Attenuated total reflectance infrared spectroscopic data were collected from 250 cm^{-1} to 6,000 cm^{-1} for **Ce1** and **Ce2** with meaningful data in the regions of 500 – 1,500 cm^{-1} and 2,400 – 3,500 cm^{-1} shown in Supplementary Figures 21 and 22. Since the only difference between **Ce1** and **Ce2** is a 4-nitrophenyl branch of the 4' carbon in the terpyridine ligand, it is expected that the IR spectroscopy should be quite similar. This is indeed found in the regions of ATR-IR spectra in both the regions of 500 – 1,500 cm^{-1} and 2,400 – 3,500 cm^{-1} although the peaks have a higher signal in **Ce2**. There is an extra couple of peaks centered at 800 cm^{-1} which correspond to the bending of a para-substituted phenyl ring.

2. SUPPLEMENTARY METHODS

2.1 Experimental

2.1.1 Synthesis and Crystal Structure

Synthesis of 1. An aqueous solution of 10% NaOH (2.5 mL) was added to a suspension of 6.25 g (41.4 mmol) of 4-nitrobenzaldehyde in 50 mL of ethanol. The resulting mixture cooled at 0 °C. Then 5.0 g (41.2 mmol) of 2-acetylpyridine was added dropwise for 3 h. The solution was stirred at 0 °C for 2 h. The precipitate formed was collected by filtration and washed with ethanol. Yield 6.91 g (66%).

Synthesis of 2. To a solution of 2 g (15.6 mmol) of 2-acetylpyridine in 20 mL of pyridine 4.60 g (17.6 mmol) I_2 was added and heated at 100 °C under N_2 for 3 h. The mixture was then cooled at room temperature and filtered off and washed with ether. The dry solid was then washed with ethanol. A black solid was obtained. It was immediately used in the next synthetic step because it is sensitive to ambient conditions. Yield 4.03 g (75%).

Synthesis of 3 (terpy*). To a solution of 10% dry ammonium acetate in 20 mL of ethanol, 1 g (3 mmol) of (2) and 0.78 g (3 mmol) (1) were added. The mixture was refluxed for two days, the solvent removed, and the product used without further purification. Yield 1.00 g (93%).

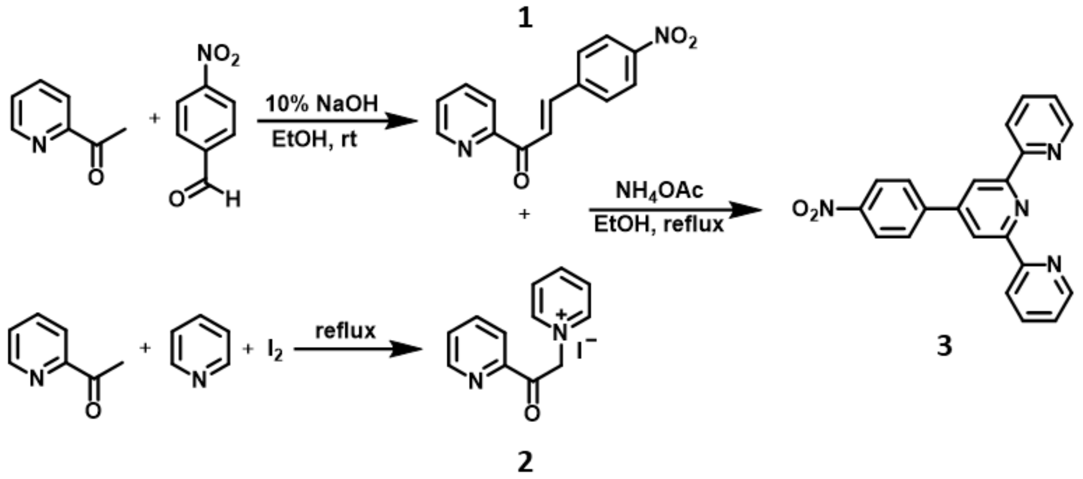

Scheme 1. Synthetic route of terpy*.[24,25]

Synthesis of metal complexes. *Caution!* ^{249}Bk *($t_{1/2}$ = 320 d; specific activity = 1.6 × 10^3 Ci/g) undergoes β-decay to* ^{249}Cf *($t_{1/2}$ = 351 y; specific activity = 4.1 Ci/ g), in addition to a small (0.1%) α-decay to* ^{245}Am*, which rapidly β-decays to* ^{245}Cm*.* ^{249}Cf *represents a significant external hazard from its intense γ-ray emission giving off energies greater than >300 keV. All studies with transuranium elements were conducted in a radiologic laboratory. Researchers were protected with moveable lead walls, lead bricks, thick lead sheets, respirators, and long lead aprons.*

Ce(terpy*)$(NO_3)_3(H_2O)\cdot(OC_4H_8)$ (Ce1). $Ce(NO_3)_3\cdot 6H_2O$ was purchased from Sigma Aldrich and used without further purification. Elemental Analysis data were collected by Midwest Microlab. 78.3 mg (0.180 mmol) $Ce(NO_3)_3\cdot 6H_2O$ and 53.1 mg (0.150 mmol) terpy* were dissolved in 10 mL of tetrahydrofuran (THF) in a 20 mL glass vial. 6 mL of acetone was layered on top of the solution. Gold crystals of **Ce1** (92.5 mg, 0.120 mmol, 80.0%) suitable for single crystal X-ray diffraction were obtained the following day. Anal. Cald. For $C_{25}CeH_{24}N_7O_{13}$: C, 38.96; H, 3.14; N, 12.72. Found: C, 39.03; H, 3.14; N, 12.64.This synthesis can be reliably scaled down to a 5-20 μmol scale . Crystal data and structure refinement as well as the bond lengths for **Ce1** may be found in Supplementary Tables 9 and 10. NMR spectroscopy could not be obtained due to solubility issues in common deuterated solvents such as *d*8-tetrahydrofuran, *d*7-ethanol, or *d*3-acetonitrile.

Bk(terpy*)$(NO_3)_3(H_2O)\cdot$(THF) (Bk1). $^{249}Bk(NO_3)_3\cdot xH_2O$ was prepared by adding dropwise 1 mL of 0.15 M NH_4OH to a mixture of ^{249}Cf and 2 mg (0.008 mmol) of ^{249}Bk in 2 mL of

ultrapure water in a 15 mL centrifuge tube. Once there was no evidence of precipitation of $^{249}Cf/Bk(OH)_3$, 30% by weight hydrogen peroxide solution was added dropwise until the lime green solution began to turn orange from the oxidation of $^{249}Bk^{3+}$ to $^{249}Bk^{4+}$. A finely divided brick-red $^{249}Bk(OH)_4$ solid settled overnight at the bottom of the tube leaving a green Cf^{3+} supernatant. The ^{249}Cf solution was carefully removed leaving the brick-red $^{249}Bk(OH)_4$ solid. This solid washed with DI water and then dissolved in 2 mL of 6 M nitric acid and transferred to a 20 mL glass vial. This solution was then dried to a residue in a box furnace at 170℃. The resultant light green solid was then re-dissolved in 2 mL of 6 M nitric acid and dried down two more times to ensure full conversion to $^{249}Bk(NO_3)_3 \cdot xH_2O$. The $^{249}Bk(NO_3)_3 \cdot xH_2O$ was subsequently dissolved in 1 mL THF. A solution of 2.8 mg (0.008 mmol) terpy* dissolved in 2 mL of THF was added to the ^{249}Bk solution. About 0.5 mL of acetone was layered on top of the THF solution. Amber crystals of **Bk1** suitable for single crystal X-ray diffraction were obtained five days later. Crystal data and structure refinement as well as the bond lengths for **Bk1** may be found in Supplementary Tables 11 and 12.

Ce(terpy)(NO$_3$)$_3$(H$_2$O)·(OC$_4$H$_8$) (Ce2). $Ce(NO_3)_3 \cdot 6H_2O$ and 2,2′:6′,2″-terpyridine (terpy) were purchased from Sigma Aldrich and used without further purification. Elemental Analysis data were collected by Midwest Microlabs. 55.0 mg (0.127 mmol) of $Ce(NO_3)_3 \cdot 6H_2O$ and 28.0 mg (0.120 mmol) of terpy were dissolved in 3 mL of THF in a 6 mL glass vial. The following day bright yellow crystals (76.5 mg, 0.117 mmol, 97.5%) suitable of single crystal X-ray diffraction were obtained. Anal. Cald. For $C_{19}CeH_{21}N_6O_{11}$: C, 35.13; H, 3.26; N, 12.94. Found: C, 34.63; H, 2.90; N, 13.31. The structure and associated plane comprised of the terpy ligand and a nitrate molecule may be seen in Supplementary Fig 20. Additionally, the crystal data and structure refinement as well as the bond lengths for **Ce2** may be found in Supplementary Tables 13 and 14. NMR spectroscopy could not be obtained due to solubility issues in common deuterated solvents such as *d*8-tetrahydrofuran, *d*7-ethanol, or *d*3-acetonitrile.

Single Crystal X-ray Diffraction. Single crystals of each compound were deposited on Mitogen mounts using Immersion Oil. The crystals were centered with a Bruker D8 Quest X-ray diffractometer equipped with an IμS X-ray source (Mo K_α, λ = 0.71073 Å) conjoined with a digital camera. Low temperature (28 K) data collections were achieved using an Oxford Cryostream N-Helix. The unit cells were determined with APEXIII software. The structures were determined using Olex[2] equipped with the SHELXTL program suite.

2.1.2 Magnetism

DC magnetic susceptibility χ = M/H measurements were carried out at temperatures T = 1.8 – 300 K using a Quantum Design VSM Magnetic Property Measurement System (Supplementary Fig. 7). Collections of small single crystalline samples of the La and Ce containing analogues were enclosed in gel-caps that were suspended in a straw for the measurement. The sample masses were 33.9 and 12.1 mg and measurements were performed under an applied magnetic field of H = 5 kOe and 1 kOe for the La and Ce analogues, respectively. The magnetic contribution of the gelcap was also measured under *H* = 1 kOe. The resulting raw magnetic field normalized magnetization data are shown in Supplementary Fig. 7b. In order to isolate the magnetic contributions of the La and Ce compounds, the gel cap contribution was subtracted from the total magnetic signal (see insets to Supplementary Fig. 7b). Finally, a Curie-Weiss fit to the data was done for the cerium containing compound for a data set where the weakly magnetic lanthanum compound χ was subtracted (inset Supplementary Fig. 7a), which yields an effective magnetic moment $\mu_{eff} \approx 2.4\ \mu_{eff}$/Ce that is consistent with expectations for trivalent cerium ($\mu_{eff,Hund's\ rules} \approx 2.54\ \mu_{eff}$/Ce).

2.1.3 Collision-Induced Dissociation Mass Spectrometry-Gas Phase Experimental Method

Gas-phase experiments were performed using an Agilent 6340 quadrupole ion trap (QIT) mass spectrometer. The instrument and general experimental approach have been described elsewhere;[8,11]only details specific to these experiments are included here. Cation complexes $[M(L)_n(NO_3)_2]^+$ where M = Ce or Eu, L = terpy or terpy*, and n = 1 or 2, were produced by electrospray ionization of tetrahydrofuran solutions containing 200 μM of $M(NO_3)_3$, and 500 μM of terpy or terpy*. The cation complex of interest was isolated in the QIT for specific isotopes ^{140}Ce or ^{151}Eu, and isotopomers terpy = $^{12}C_{15}{}^{1}H_{11}{}^{14}N_3$ (233 Da) or terpy* = $^{12}C_{21}{}^{1}H_{14}{}^{14}N_4{}^{16}O_2$ (354 Da), and then subjected to collision induced dissociation (CID). During CID an ion is resonantly excited and undergoes multiple energetic collisions with helium to ultimately induce dissociation. Dissociation products and yields are identified by mass-selective ejection from the QIT, followed by ion detection. The CID energy under these conditions is not quantified, but it increases with increasing

the instrumental parameter referred to as “CID voltage”. Also, for two ions of similar mass (m/z), the same instrumental conditions, including CID voltage, result in comparable excitation such that a higher CID yield indicates a more efficient reaction. For a simple ligand-elimination process that can be considered to be essentially barrierless, higher efficiency indicates a lower energy process, which indicates lower endothermicity and lower ligand binding energy. The mass spectra were acquired using the following instrumental parameters in the positive ion mode: solution flow rate, 60 μL/min; nebulizer gas pressure, 15 psi; capillary voltage, -3500 V; end plate voltage offset, −500 V; dry gas flow rate, 2 L/min; dry gas temperature, 325 °C; capillary exit, 150.1 V; skimmer, 40.0 V; octopole 1 and 2 dc, 12.00 and 2.22 V; octopole RF amplitude, 200.0 Vpp; lens 1 and 2, −5.0 and −60.0 V; trap drive, 77.9. High-purity nitrogen gas for nebulization and drying in the ion transfer capillary was the boil off from a liquid nitrogen Dewar. The helium buffer gas pressure in the QIT was constant at $\sim 10^{-4}$ Torr.

2.1.4 Magnetic Circular Dichroism

Crystalline material of Ce1 was finely ground in a mulling agent (fluorolube) and a thin layer placed between two quartz disks which were fitted into a copper cell. The UV-visible MCD spectra were collected using a Jasco J-715 spectropolarimeter and a shielded S-20 photomultiplier tube. This instrument utilizes a modified sample compartment incorporating focusing optics and an Oxford Instruments SM4000-7T superconducting magnet/cryostat, permitting measurements from 1.6 to 290 K with magnetic fields of up to 7 T. All MCD spectra were baseline-corrected against zero-field scans.

2.1.5 Electrochemistry

10 mM $Ce(NO_3)_3$ and Terpy solutions were prepared in THF containing 100 mM tetra-n-butylammonium hexafluorophosphate ($TBAPF_6$), purchased from VWR, which served as supporting electrolyte. Upon combination in a 1:1 ratio, a 5 mM solution of the Ce1 complex formed. Voltammetry was performed within 12 hours of complex formation, since crystallization of the complex in solution was known to occur over a similar timescale. A solution containing Bk1 was similarly prepared, although the concentration was not known. The supporting electrolyte for electrochemical studies on Bk1 was 100 mM tetrabutylammonium tetrakis(3,5-bis(trifluoromethyl)phenylborate. All experiments were taken at room temperature (19.5 °C), and no effort to remove water or oxygen from the solutions was taken.

All cyclic voltammetry (CV) measurements were performed on a CH Instruments 660e potentiostat, using a 3-electrode setup. A 2 mm diameter Pt disk working electrode (CH Instruments), a Pt wire counter electrode (Alfa Aesar), and a Ag/Ag^+ pseudo-reference electrode (CH Instruments) were used. This was followed by sonication in deionized water, drying in air and then rinsing in THF. The pseudo-reference electrode comprised a Ag wire housed in a glass compartment, filled with 100 mM $AgNO_3$ in MeOH, both materials purchased from VWR. To account for variability in this variability in the potential of the pseudo-reference electrode, the ferrocene/ferrocenium redox couple (Fc/Fc^+) was used as an internal reference. A nominal concentration of Fc was added to the solution so that Fc/Fc^+ voltammograms could be recorded before and after voltammograms on the complexes. The Pt disk working electrode was sonicated in acetonitrile to remove the complex and supporting electrolyte followed by polishing in a 50 nm alumina suspension (Buehler) for two minutes between each cylic voltammogram to ensure a clean electrode surface. These cleaning steps were followed by sonication in deionized water, drying in air and then rinsing in THF. A clean, pristine electrode surface is highly important for acquiring reliable data from CVs. Oxidation (E_{anod}) and reduction (E_{cath}) potentials were estimated from the CV, with plotting and analysis performed using MATLAB.

2.1.6 EPR Spectroscopy

Samples of **Ce1** were finely ground using a mortar and pestle and loaded into a 4 mm OD suprasil screwcap EPR tube. The 5 K X-band EPR spectrum was recorded on a Bruker EMXplus spectrometer equipped with a 4119HS cavity and an Oxford ESR-900 helium flow cryostat. The instrumental parameters employed for samples are as follows: 1 mW power; time constant 41 ms; modulation amplitude 8 G; 9.40 GHz; modulation frequency 100 kHz.

2.1.7 Solid State UV-visible-NIR Spectroscopy

Single crystals of **Ce1** and **Bk1** were carefully placed in immersion oil on a glass slide and transferred to the microscope stage of a Craic Technologies 20/20 PVTM Dual Microspectrophotometer. Absorption spectra were collected from 320 nm to 1,700 nm with a 75 W Xe light source. Detector integration times were optimized for each crystal using the built-in Craic software.

2.1.8 Powder X-ray Diffraction

Single crystals of **Ce1** were ground into a fine powder, and the powder pattern was collected using a Rigaku SmartLabs SE diffractometer equipped with a Cu K_α source at 40 kV and 44 mA, divergent beam optics, and a Rigaku D/tex detector. The pattern in Supplementary Fig. 20 shows a high purity sample.

2.1.9 ATR-IR Measurements

A small amount (50-60 microcrystals) of **Ce1** and **Ce2** were measured by a JASCO 6800 FT-IR Spectrometer from 250 to 6,000 cm^{-1} at a resolution of 0.12 cm^{-1} at 144 separate scans for each spectrum.

2.2 Computational Details

2.2.1. Wavefunctions. The electronic structures of **Bk1** and **Ce1** were analyzed through the Complete Active Space Self Consistent Field (CASSCF) method Given the sensitivity of bonding and magnetism to the molecular geometry, the experimental geometries were used to obtain the wavefunctions.

The electronic structure calculations were carried out with version 8.4 of MOLCAS code.[12] The Douglas–Kroll–Hess scalar Hamiltonian[13] was employed in the calculations without spin-orbit coupling (SOC), along with all-electron ANO-RCC Gaussian-type basis sets contracted to TZP quality (Ce = 25s22p15d11f4g2h/8s7p4d3f2g1h and C, N, O = 14s9p4d3f2g/4s3p2d1f). The trial set of orbitals for the complete active space self-consistent field (CASSCF) procedure[14] was obtained from Hartree-Fock (HF) calculations. Once obtained the CASSCF molecular orbitals, dynamical electron correlation was included by the complete active space perturbation theory at second order (CASPT2).[15] SOC was treated by state interactions between the CASSCF/PT2 wave functions, using the restricted active space state interaction (RASSI) program.[16] The spin-orbit operator matrix was calculated from atomic mean-field (AMFI) spin-orbit integrals.[17] On the basis of the resulting SOC multiplets, the SINGLE_ANISO program[18] was used to compute Zeeman splitting (g-tensor) and static magnetic properties, molar magnetic susceptibility and magnetization. Additionally a one-to-one mapping of the *ab initio* results onto the classical crystal field acting on the ground multiplet was performed using the crystal-field Hamiltonian $\hat{H}_{CF} = \sum_{k,q} B_k^q \hat{O}_k^q$, where B_k^q are the parameters describing the crystal-field and $\hat{O}_k^q$ the Stevens operators.[19]

Expansions of the active spaces were performed through the inclusion of metal *d* using, as a starting point, the minimal active space wave function CAS(n,7) n=1 (Ce), 8 (Bk). The active space included the closest in energy *d* orbital resulting in CAS(n,10) n=3 (Ce), 10 (Bk) (Supplementary Figure 1 and 2) active spaces. The incorporation of only one *d* orbital was based on the splitting of around 4,200 cm^{-1} with respect to the next *d* orbital. Despite the fact that *g*-factors remained almost unchanged, the assessment of the $5f^8 \longrightarrow 5f^7\ 6d^1$ transition was not accurate because of the lack of the remaining *d* orbitals (bonding and antibonding). Then a second attempt including the *f* shell + *d* shell CAS(n,12) n=1 (Ce), 8 (Bk) was done. As expected no significant changes in the *g*-factors were observed and the assignment of the $5f^8 \longrightarrow 5f^7\ 6d^1$ transition was closer to previous values reported for Bk(III), which are not seen in the experimental UV-Vis spectrum. When ligand-based bonding or antibonding orbitals with respect to the *f*- and *d*-orbitals were attempted to be included into the active space, the complexity of the bonding pattern in these molecules made impossible to find the appropriate counterpart. As observed, the drawback of expanding the active space is that it is hard to keep the bonding/antibonding balance due to the involvement of multiple ligands and their valence orbitals. This causes that the most appropriate active spaces increase significantly in dimension making the CASSCF approximation impractical. This is probably the biggest drawback for bonding analysis and the reader must bear in mind that these results lack of electron correlation. This might be lessen by the fact that HF and post HF-derived molecular density are overlocalized by nature.[20] Thus, any degree of covalency observed from these wavefunctions would be a safe place to establish this interaction rather than approaching from an overestimation of it as would be the case of DFT densities.

2.2.2 Molecular Properties. The CASSCF wavefunctions were used for electronic structure-based analyses such as magnetism and bonding, where state-average wavefunctions were used for the former and state-specific for the latter. Thus, QTAIM and interacting quantum atom (IQA) calculations on the ground state were performed based on state-specific densities. The AIMAll software was used to perform these analyses.[21] It is important to note that the 2-electron density matrix (2EDM) is obtained from the 1EDM, and therefore introducing errors in the additivity of the individual components. Though, individual terms can be compared, the total energies should not.

Given that the ligand-field density functional theory (LF-DFT) approximation was also considered, which is only available within the ADF2019 suite [22,23], Kohn-Sham orbitals

were also calculated. The LF-DFT[24] calculations were performed using the hybrid PBE0 functional along with the STO-TZP basis set. The choice of the functional comes with the best description of the electronic structure for the ground and excited states of lanthanides (Ln) and actinides (An). It is noteworthy that for Ln, GGA functionals also provide a good description of the electronic structure, however, the actinides are more sensitive to the nature of the functional.

Finally, to help with the elucidation behind the experimental MCD spectra, simple time-dependent DFT (TD-DFT) calculations were performed. The excitation spectrum was produced at the BP86/STO-TZP level of theory given that this functional was able to reproduce the intra-ligand/charge transfer band.

3. SUPPLEMENTARY FIGURES

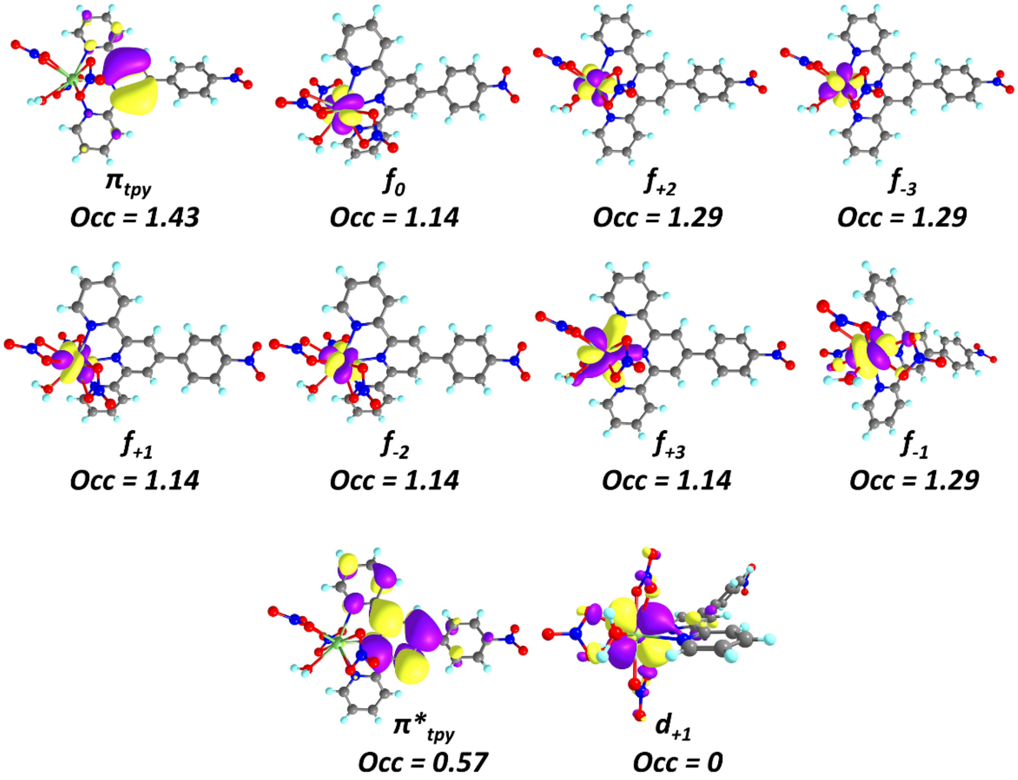

Supplementary Fig. 1 | CASSCF natural orbitals of Bk1. Graphical depiction of the natural molecular orbital obtained from a CAS(10,10) wavefunction. Labels were assigned based on the largest *f*-component contribution to the molecular orbital. The order of the orbitals does not necessarily correlate with their corresponding energies. The occupations correspond to those of the ground state.

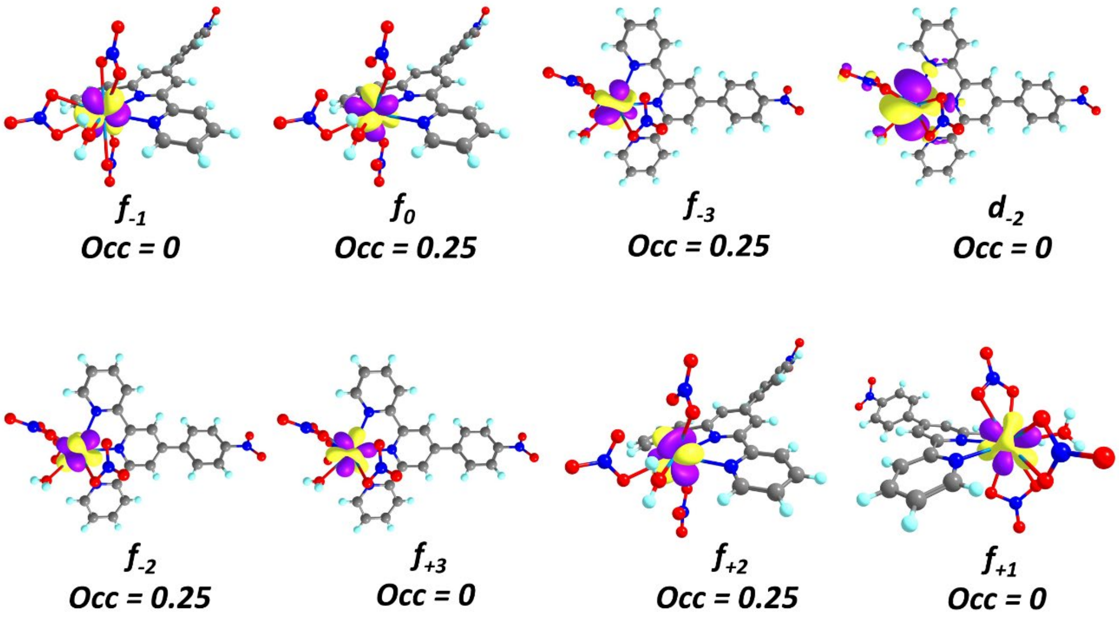

Supplementary Fig. 2 | CASSCF natural orbitals of Ce1. Graphical depiction of the natural molecular orbital obtained from a CAS(1,8) wavefunction. Labels were assigned based on the largest *f*-component contribution to the molecular orbital. The order of the orbitals does not necessarily correlate with their corresponding energies. The occupations correspond to those of the ground state.

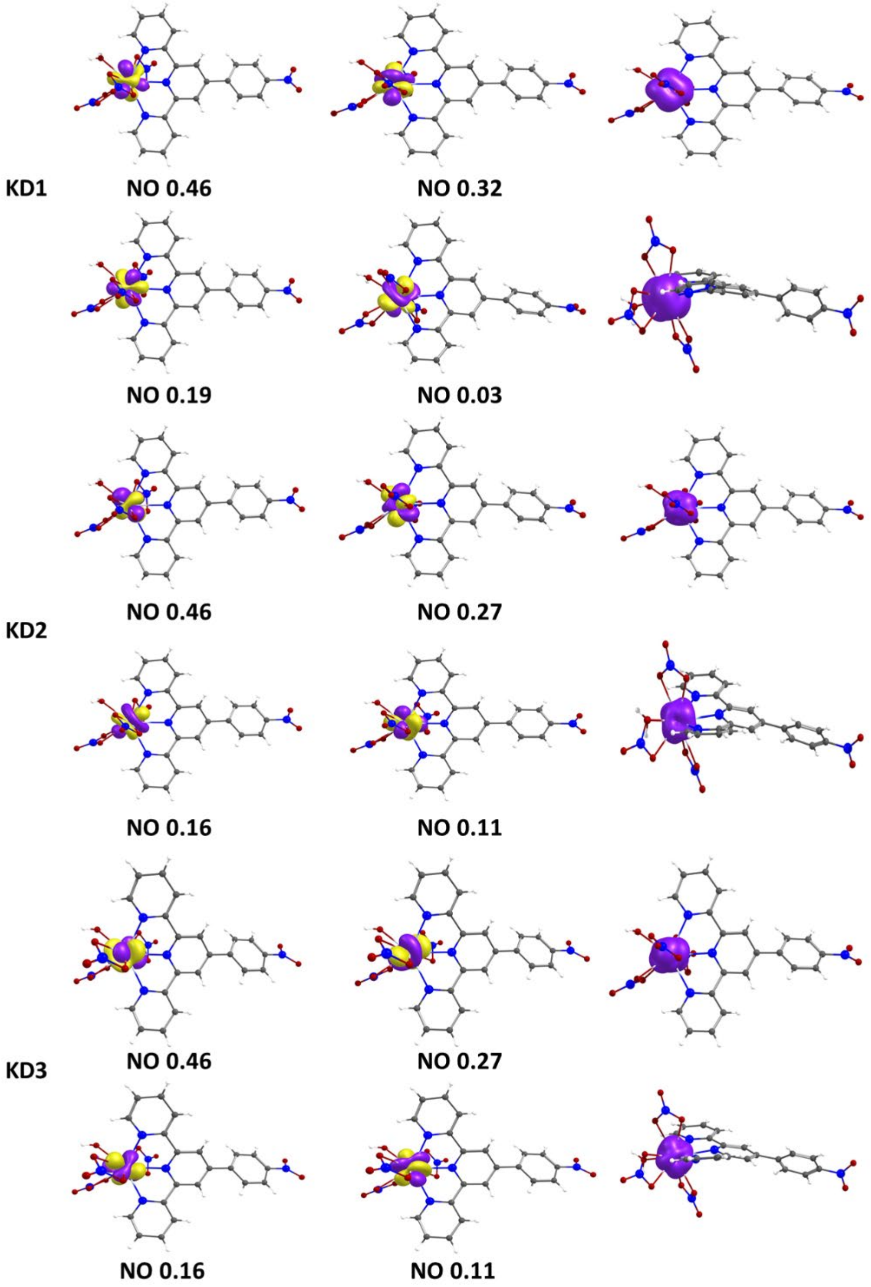

Supplementary Fig. 3 | Selected spin-orbit natural orbitals (SONOs) for Ce1. SONOs include their occupation numbers. On the far right it is shown the 4*f* electron density from two different perspective (isosurface value ± 0.03 a.u.) for the three Kramers doublets (KD) derived from the $^2F_{5/2}$ multiplet of **Ce1**. The electron density surfaces are made up of natural orbitals whose occupation numbers add up to the total number of electrons. These calculations show the asphericity of the electron density with less electron density along the x-magnetic axis with respect

to the yz-plane in KD1 and KD2, whereas for KD3 the asphericity of electron density is concentrated in the xy-plane in KD3.

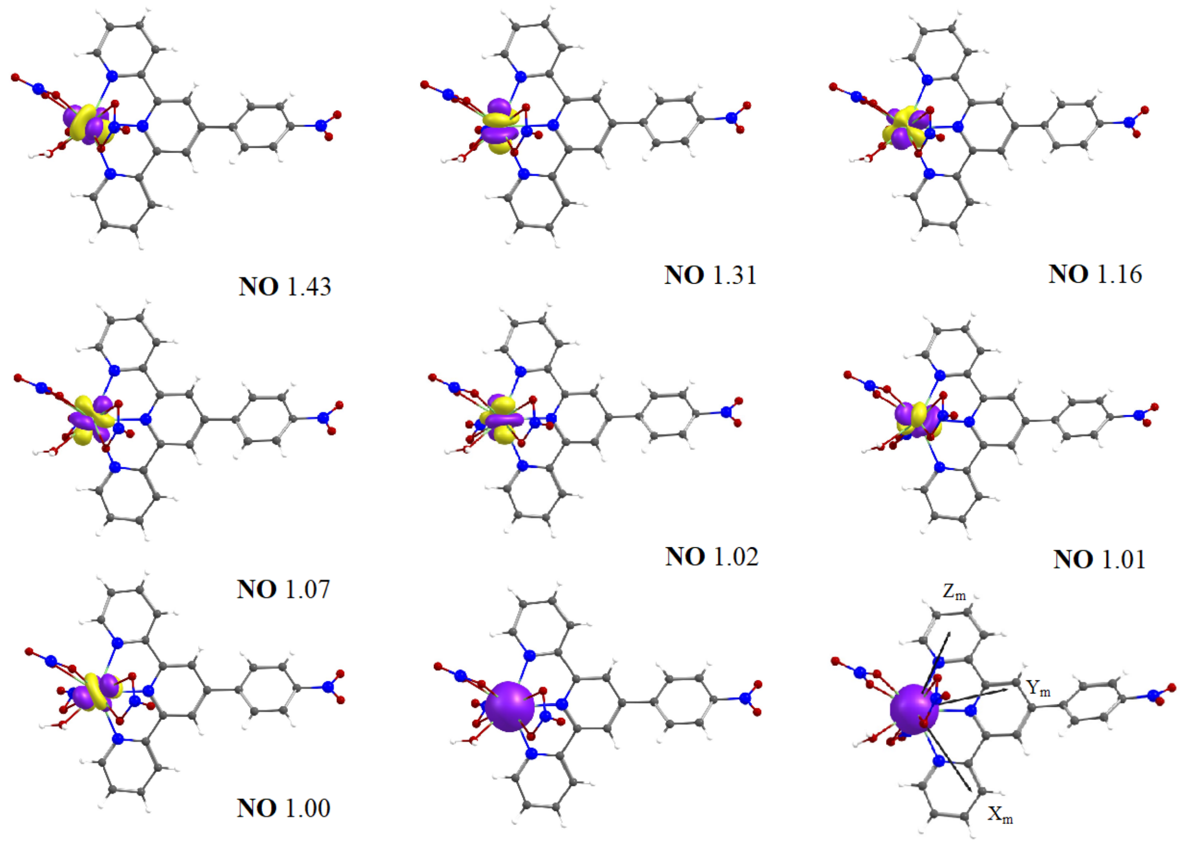

Supplementary Fig. 4 | Selected spin-orbit natural orbitals (SONOs) for Bk1. SONOs include their corresponding occupation numbers. The 5*f* electron density (isosurface value ± 0.03 a.u.) of the ground state derived from the $^7F_6$ multiplet of **Bk1** is shown at the bottom mid and bottom left of the figure. The accidental degenerate ground state shows an easy axis oriented along the axis of the *quasi-ellipsoid* determined by the oblate shape of the 5*f* electron density (see figure bottom right).

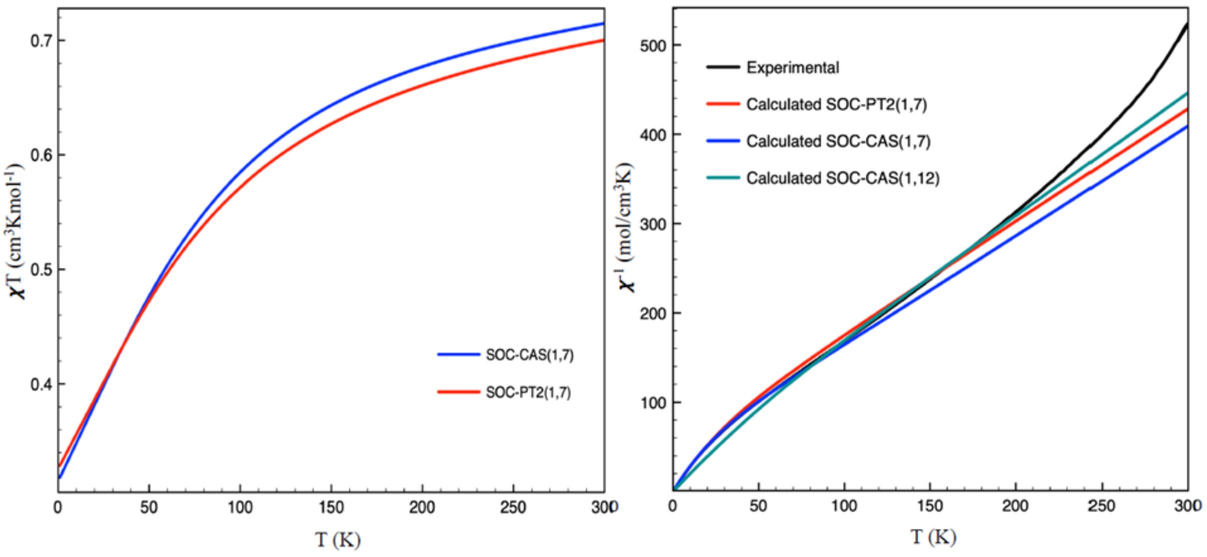

Supplementary Fig. 5 | Calculated magnetic susceptibility (χT and χ^{-1}) for Ce1. Calculated dependence of the χT and χ^{-1} with temperature at SOC-CASSCF and SOC-NEVPT2 level of theory for **Ce1**. A Boltzmann distribution analysis shows that at room temperature the three Kramers doublets (KDs) derived from $^2F_{5/2}$ multiplet are populated with a relative distribution of 56% KD1, 30% KD2 and 14% KD3.

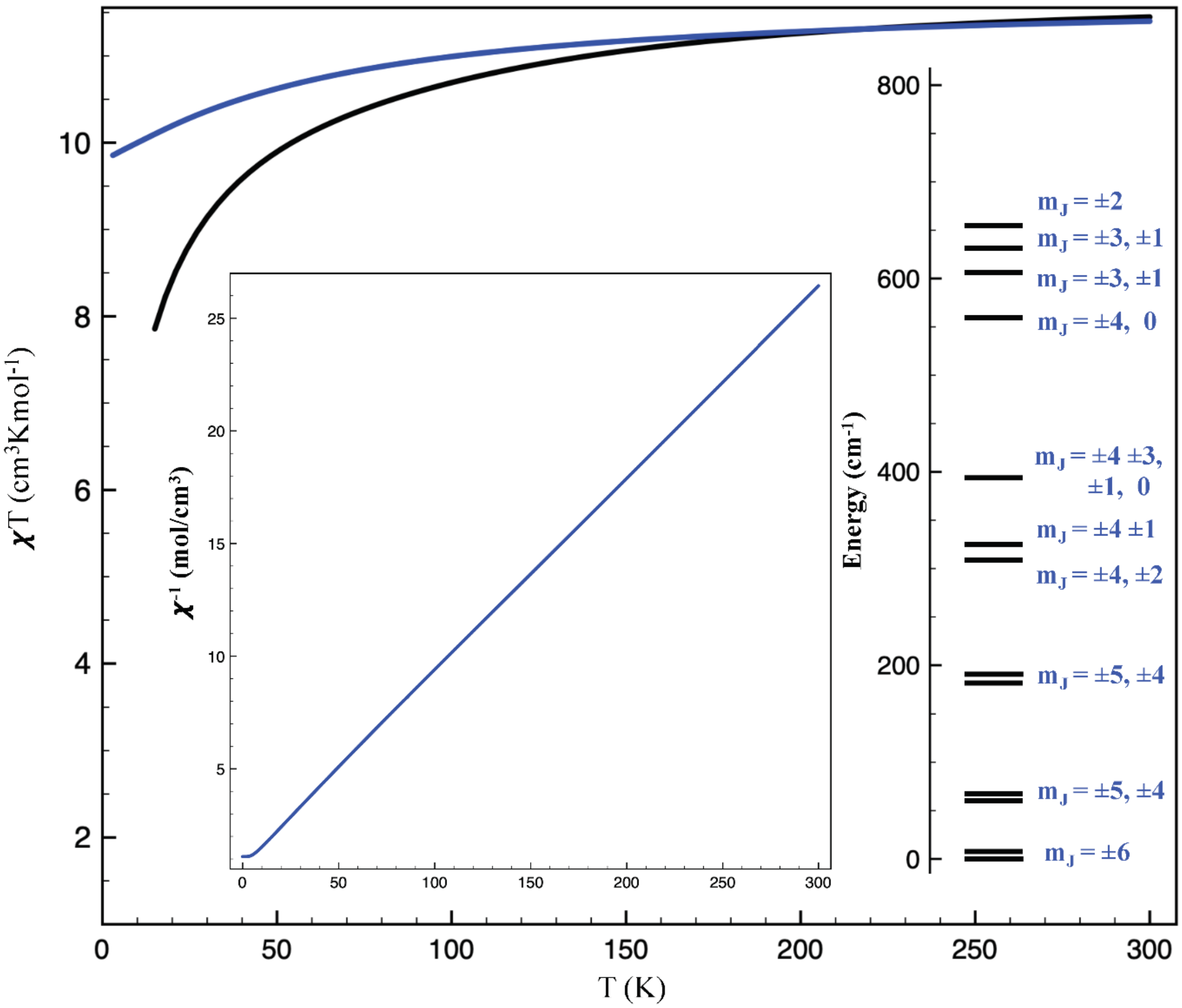

Supplementary Fig. 6 | Calculated magnetic susceptibility (χT and χ-1) for Bk1. Predicted dependence of the χT and χ^{-1} with temperature derived from SOC-CAS(8,7) (black curve) and SOC-CAS(8,12) (blue curve) wavefunctions for **Bk**1. χT values at room temperature are in the range observed in other Bk(III) complexes[3] with expected temperature dependence in all the studied range. The inset corresponds to the energy diagram of the ground multiplet in terms of the m_J compositions.

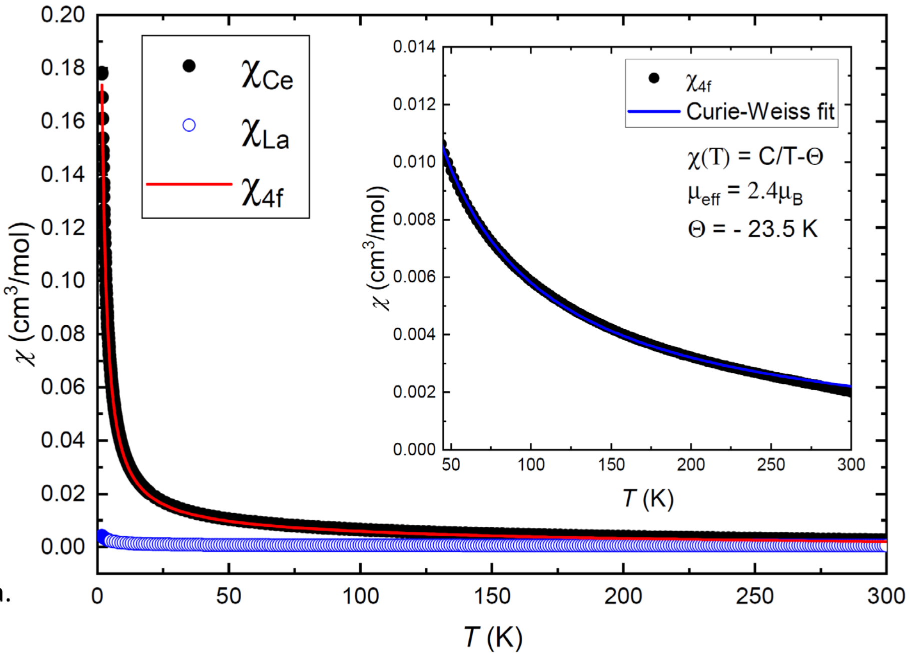

a.

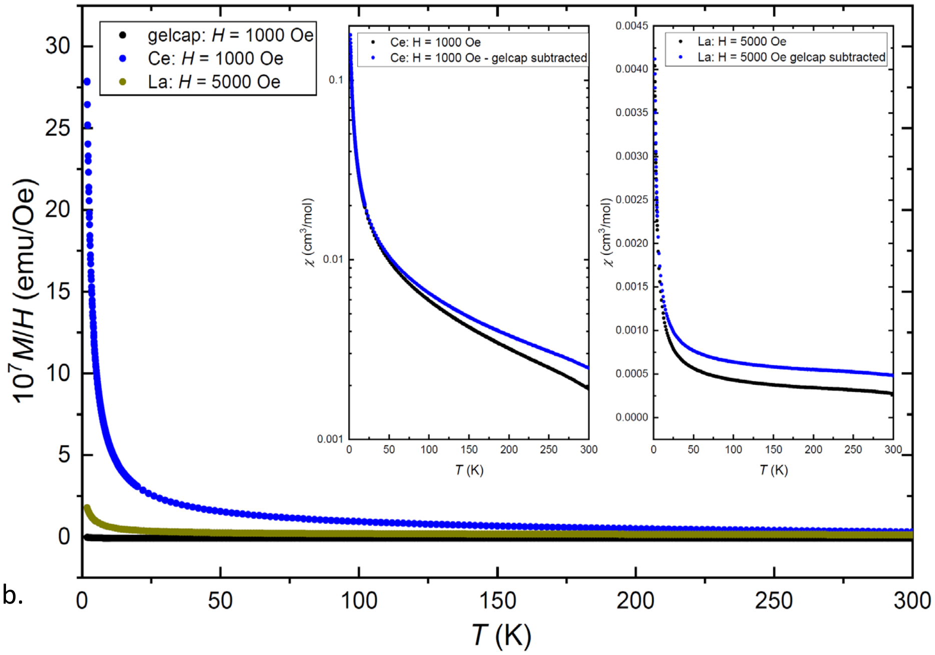

b.

Supplementary Fig. 7 | Temperature dependent DC magnetic susceptibility χ = M / H. 7.a The temperature dependent magnetic susceptibility for the nonmagnetic La (χ_{La}) and magnetic Ce (χ_{Ce}) analogues are shown in the left panel. In order to isolate the component of the magnetic

response that is due solely to the 4*f* electrons from the cerium ions (χ_{4f}, red line in main panel), χ_{La} is subtracted from χ_{Ce}. In inset a Curie-Weiss fit to $\chi_{4f}(T)$ is shown. This fit yields an effective magnetic moment μ_{eff} = 2.4 μ_B/Ce, which is slightly reduced from the expected value for trivalent cerium μ_{eff} = = 2.54 μ_B/Ce. It is also seen that the Curie-Weiss temperature Θ = -23.5 K, revealing that there is an antiferromagnetic exchange interaction between the cerium ions. **7b.** Comparison between the temperature dependent magnetic susceptibility $\chi = M/H$ where *M* is the magnetization and the applied magnetic field *H* for the cerium and lanthanide compounds. The red line is the difference between χ_{Ce} and v_{La}, which represents $\chi_{4f,}$ the isolated contribution from the cerium outer shell electrons. The inset shows a Curie-Weiss fit to χ_{4f}, where the effective magnetic moment μ_{eff} = 3.4 μ_B (consistent with trivalent cerium) and the Curie-Weiss temperature Q = -23.5 K are obtained.

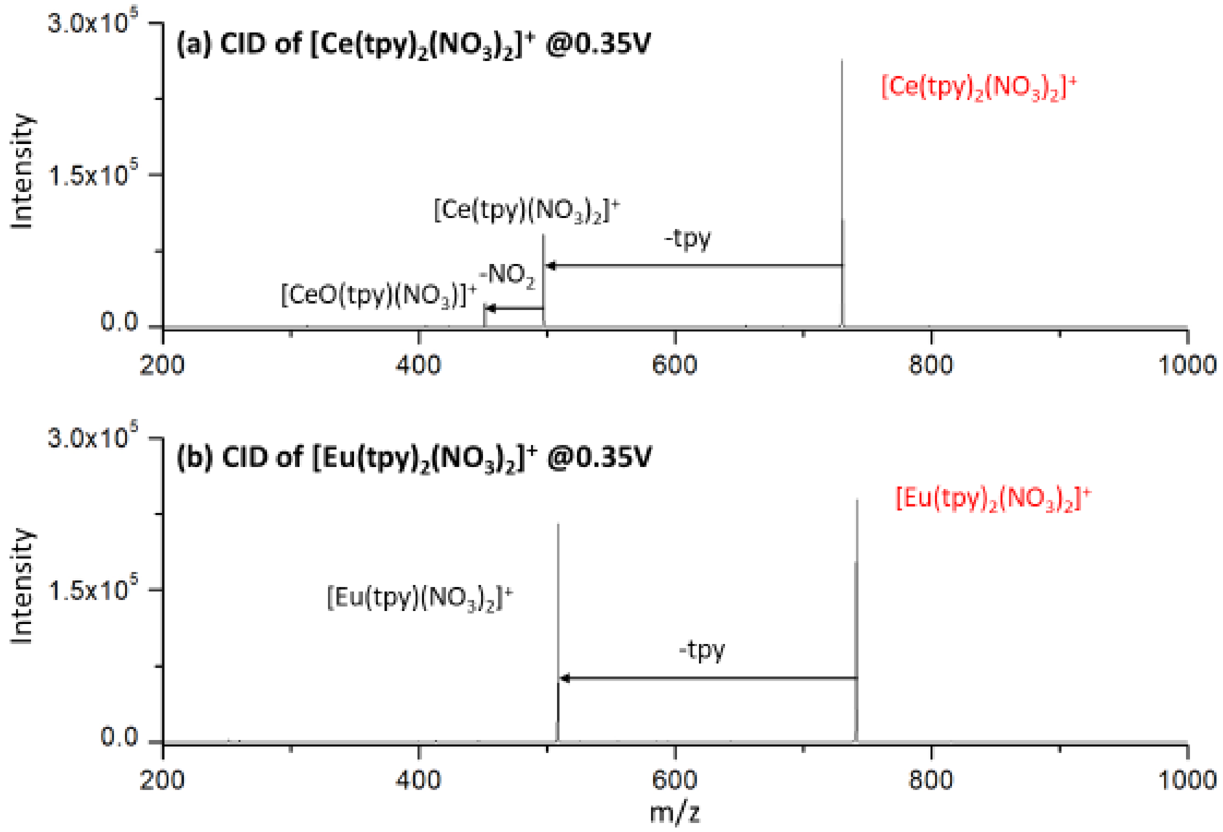

(a) CID of [Ce(tpy)2(NO3)2]+ @0.35V
[Ce(tpy)2(NO3)2]+
[Ce(tpy)(NO3)2]+
-tpy
-NO2
[CeO(tpy)(NO3)]+
Intensity
3.0x10^5
1.5x10^5
0.0
200
400
600
800
1000
(b) CID of [Eu(tpy)2(NO3)2]+ @0.35V
[Eu(tpy)2(NO3)2]+
[Eu(tpy)(NO3)2]+
-tpy
m/z

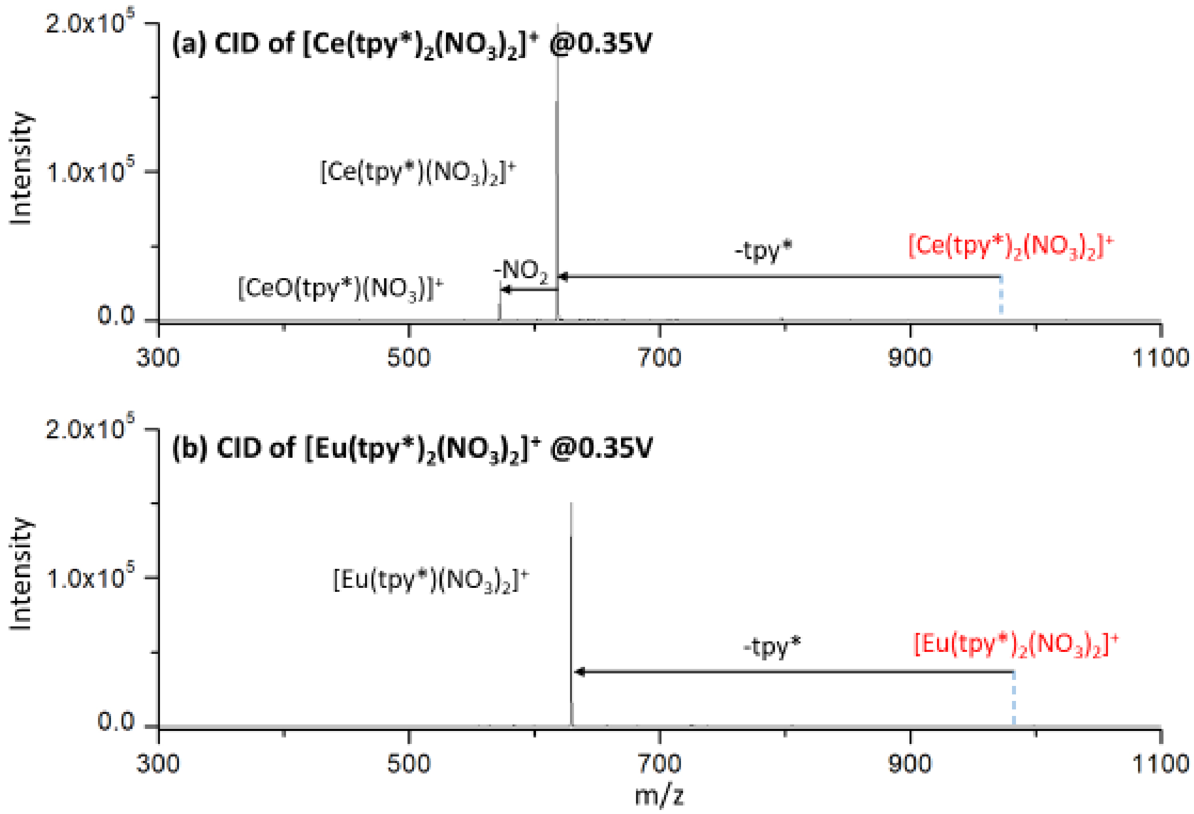

Supplementary Fig. 9 | CID mass spectra of [M(terpy*)$_2$(NO$_3$)$_2$]$^+$ for (a) M = Ce and (b) M = Eu, both at a nominal CID voltage of 0.35 V. Complete fragmentation of both complexes at the same CID voltage as employed in Supplementary Figure 8, where fragmentation is incomplete, indicates more facile elimination of L = terpy* versus L = terpy from [M(L)$_2$(NO$_3$)$_2$]$^+$, for both M = Ce and M = Eu.

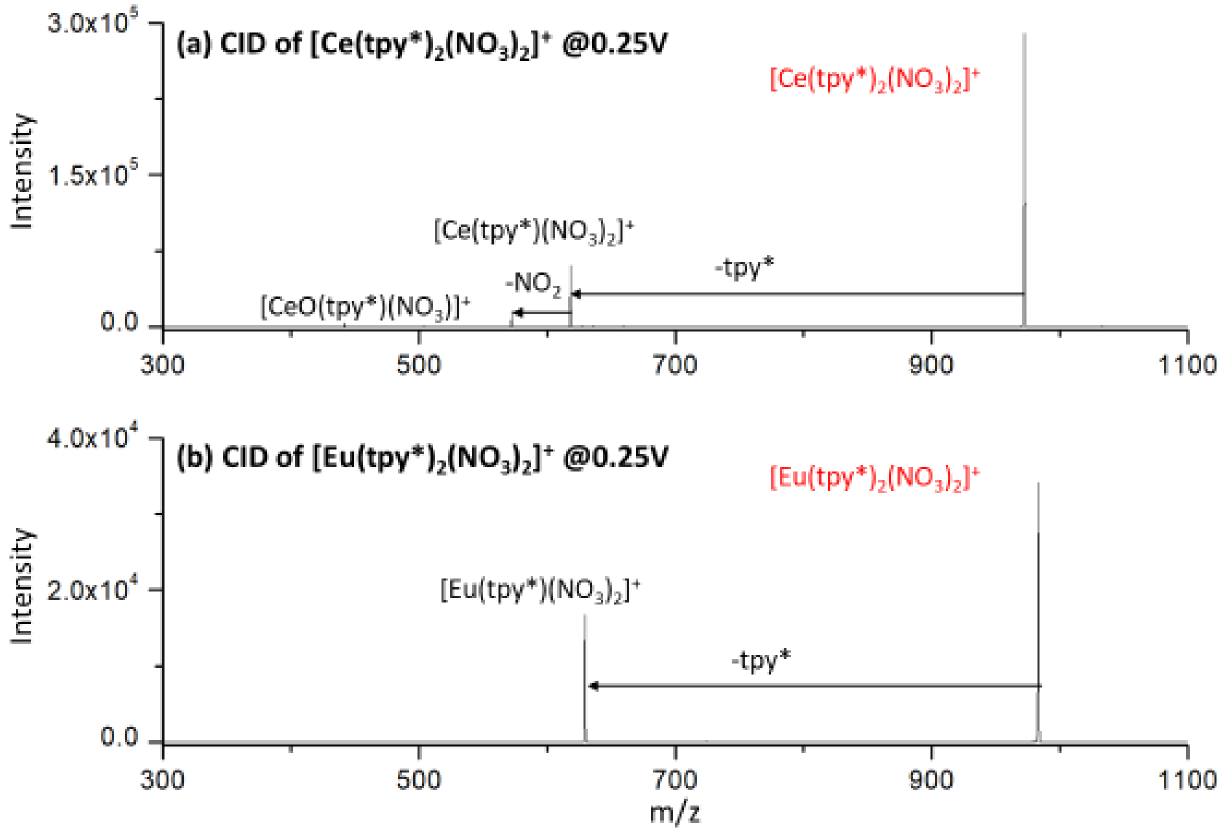

Supplementary Fig. 10 | CID mass spectra of [M(terpy*)$_2$(NO$_3$)$_2$]$^+$ for (a) M = Ce and (b) M = Eu, both at a nominal CID voltage of 0.25 V. More facile elimination of terpy* from the Eu complex is indicated by a higher product yield for M = Eu (33% yield) versus M = Ce (20% yield) under the same CID conditions. Another difference between the two complexes is the appearance of secondary NO_2 elimination from the Ce complex to oxidize from Ce^{III} in the precursor complex to Ce^{IV} in [CeO(terpy*)(NO$_3$)]$^+$.

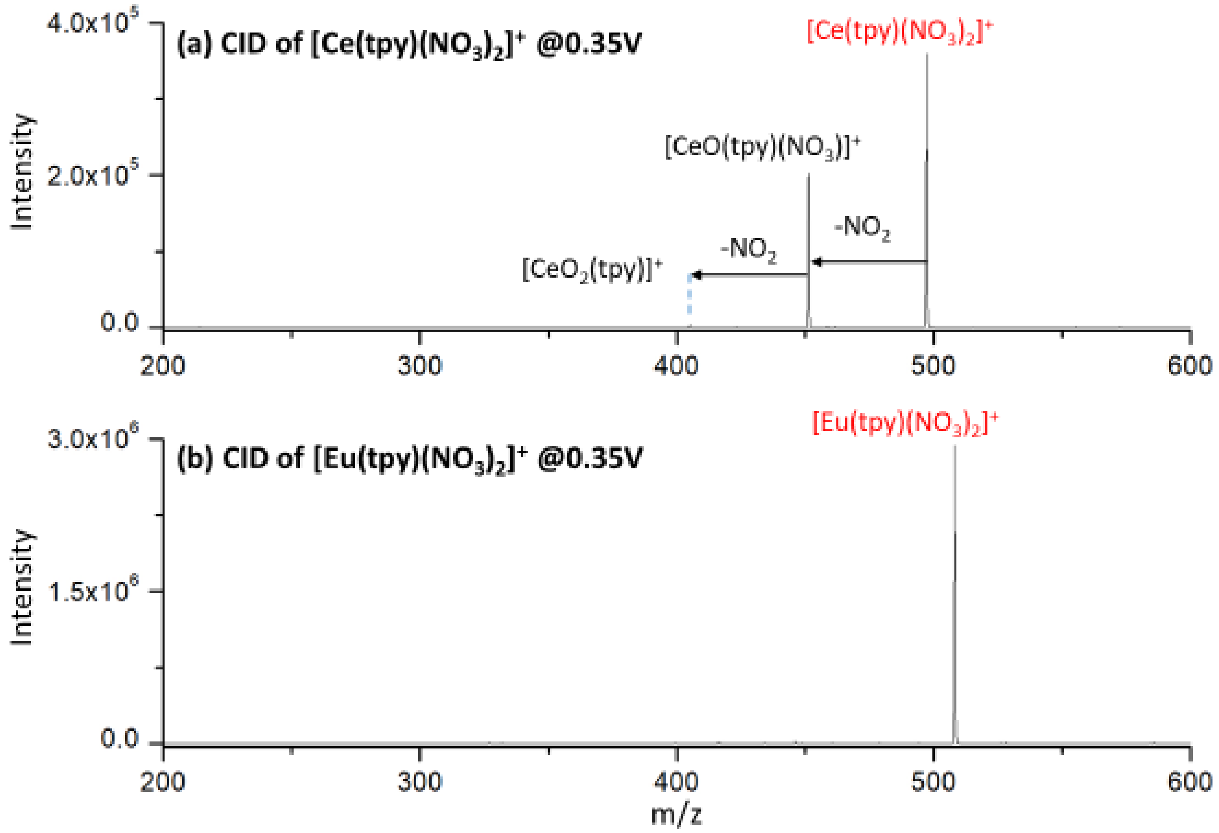

Supplementary Fig. 11 | CID mass spectra of [M(terpy)(NO3)2]+ for (a) M = Ce and (b) M = Eu, both at a nominal CID voltage of 0.35 V. In contrast to CID of the corresponding di-ligated complexes $[M(terpy)_2(NO_3)_2]^+$ under the same conditions (Supplementary Figure 8), no terpy ligand elimination is detected. The only identified CID processes are elimination of one and two NO_2 from the M = Ce complex to yield $[Ce^{IV}O(terpy)(NO_3)]^+$ and very minor $[CeO_2(terpy)]^+$, where the oxidation state in the latter species is indeterminate.

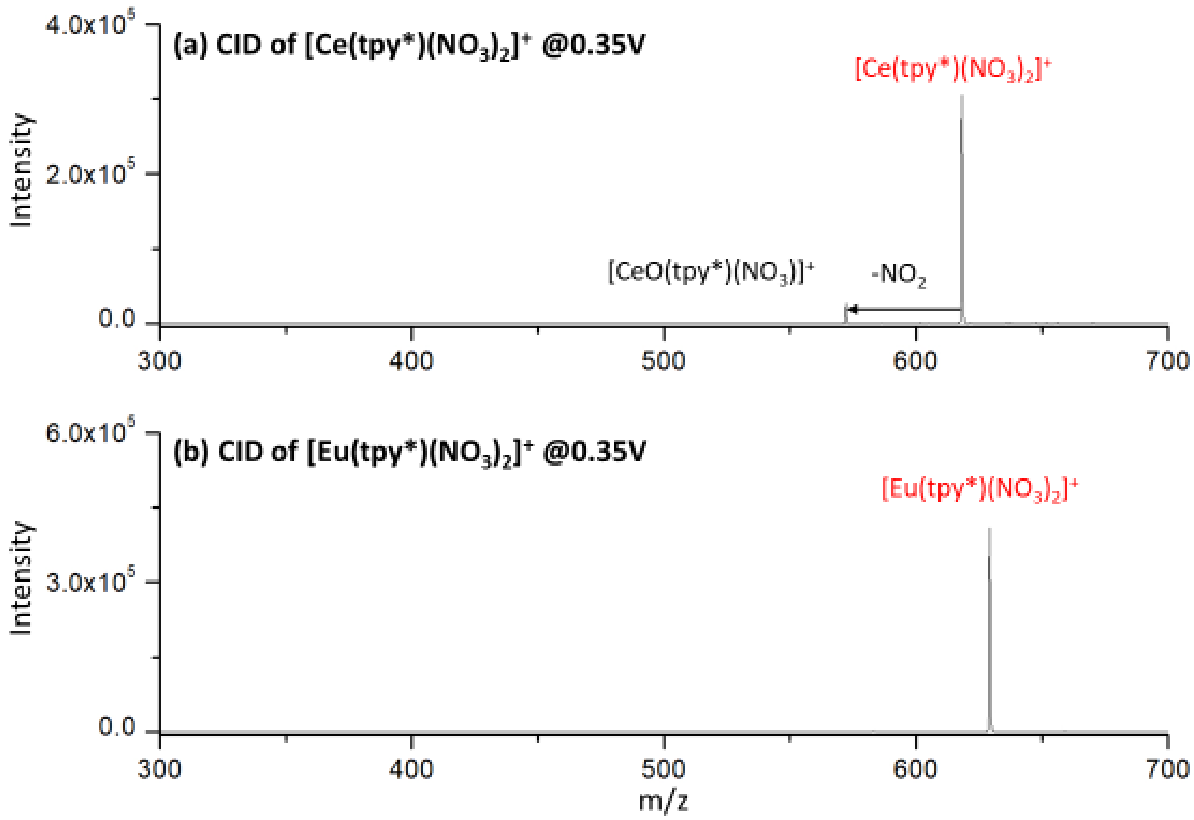

Supplementary Fig. 12 | CID mass spectra of [M(terpy*)$(NO_3)_2$]$^+$ for (a) M = Ce and (b) M = Eu, both at a nominal CID voltage of 0.35 V. In contrast to CID of the corresponding di-ligated complexes [M(terpy*)$_2$$(NO_3)_2$]$^+$ under the same conditions (Supplementary Figure 9), no terpy* ligand elimination is detected. The only identified CID processes are elimination of one NO_2 from the M = Ce complex to yield [Ce^{IV}O(terpy*)(NO_3)]$^+$.

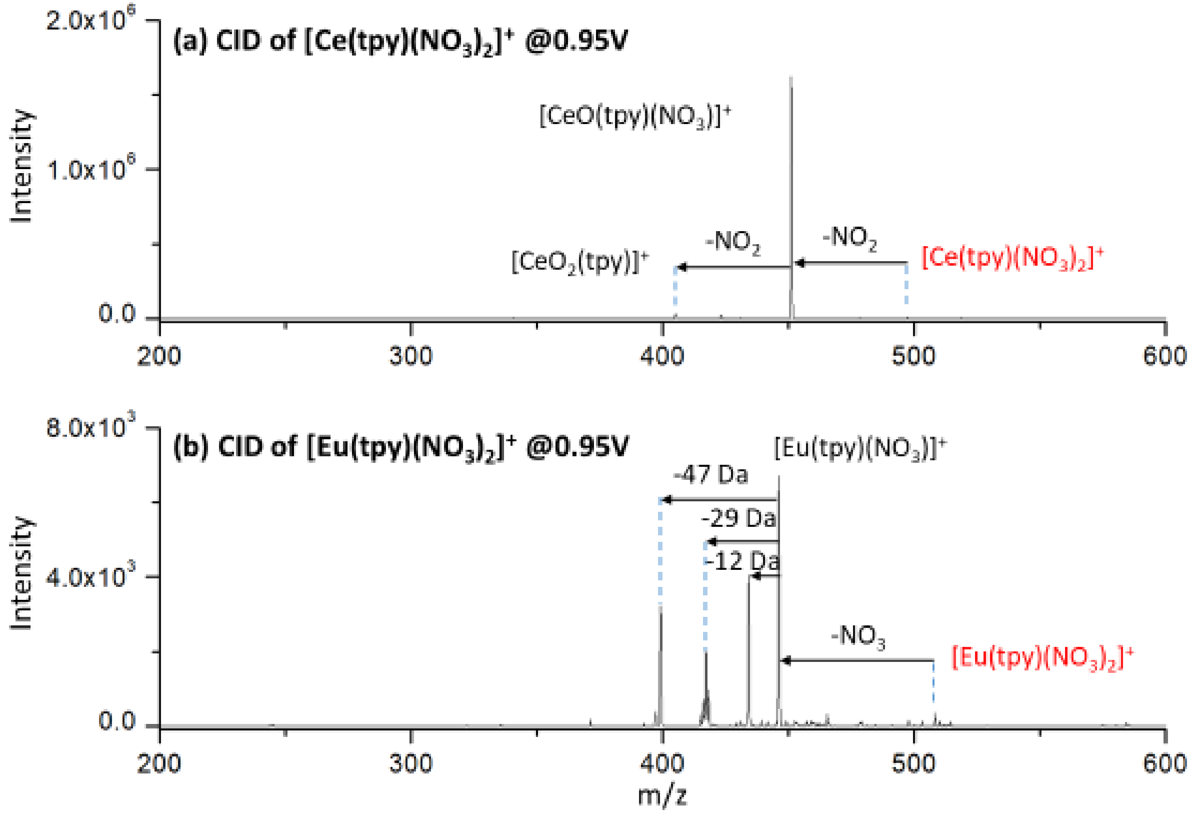

Supplementary Fig. 13 | CID mass spectra of $[M(terpy)(NO_3)_2]^+$ for (a) M = Ce and (b) M = Eu, both at a nominal CID voltage of 0.95 V, with essentially complete fragmentation of the precursor complexes. This is the same experiment as in Supplementary Figure 11 but at a significantly higher CID energy. The primary process in (a) is elimination of NO_2 to yield $[Ce^{IV}O(terpy)(NO_3)]^+$, with minor secondary elimination of another NO_2 to yield $[CeO_2(terpy)]^+$ in which the cerium oxidation state is indeterminate. The primary process in (b) is loss of 64 m/z, which is assigned as NO_3, to yield $[Eu^{II}(terpy)(NO_3)]^+$ in which Eu(III) has been reduced to Eu(II). Also apparent in (b) are fragmentations corresponding to additional losses of 12 Da, 29 Da and 47 Da, which are unassigned but are presumably due to cleavage of the terpy ligand. A remarkable feature of both of these CID spectra, (a) and (b), is that no loss of intact ligand terpy is detected, this in contrast to results for $[M(terpy)_2(NO_3)_2]^+$ in Supplementary Figure 8 where terpy loss is the dominant process at a much lower CID energy of 0.35 V. These comparative results indicate stronger binding of the terpy ligand in $[M(terpy)(NO_3)_2]^+$ versus those in $[M(terpy)_2(NO_3)_2]^+$.

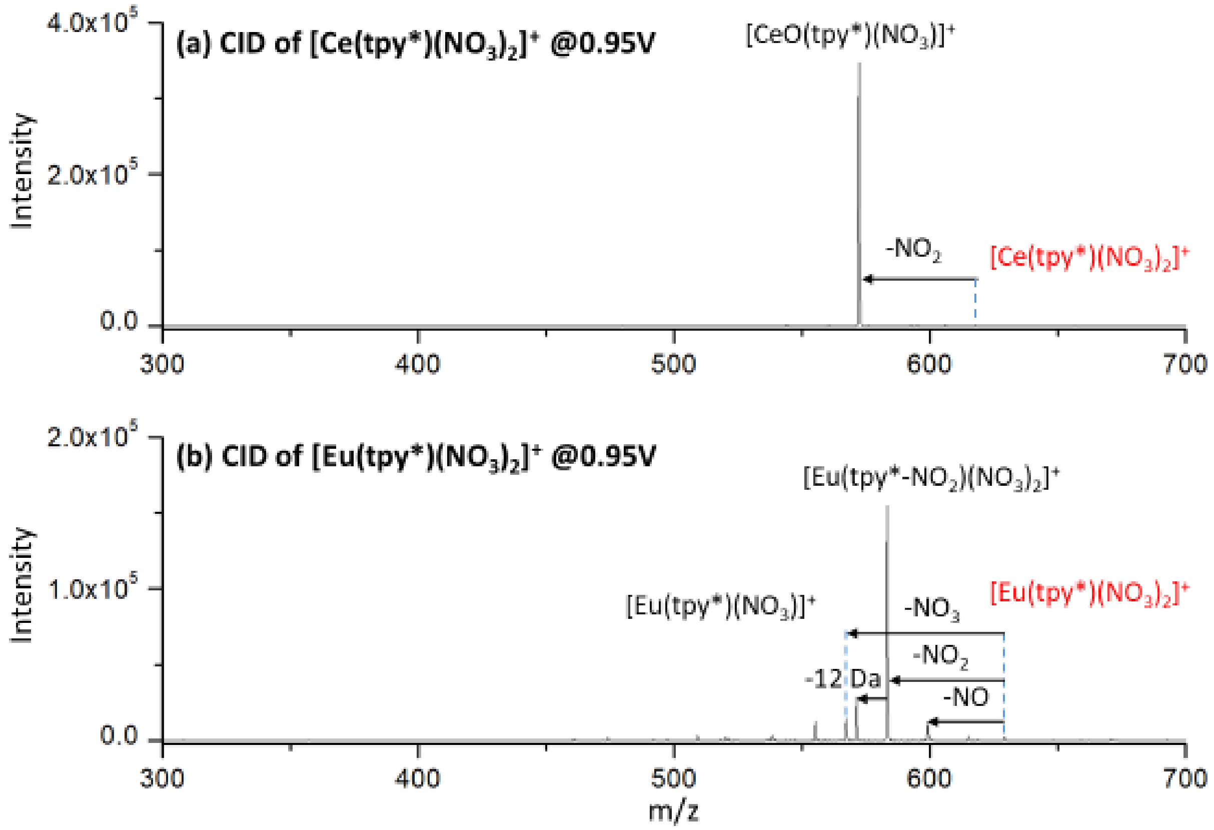

Supplementary Fig. 14 | CID mass spectra of $[M(terpy^*)(NO_3)_2]^+$ for (a) M = Ce and (b) M = Eu, both at a nominal CID voltage of 0.95 V. This is the same experiment as in Supplementary Figure 12 but at a significantly higher CID energy. The only process apparent in (a) is elimination of NO_2 to yield $[Ce^{IV}O(terpy^*)(NO_3)]^+$, in analogy with the results in Supplementary Figure 13 (a) for the corresponding terpy complex. The dominant process in (b) is elimination of NO_2. Based on the complete absence of this channel in Figure 13 (b) for the corresponding terpy complex, it is assigned here to elimination of NO_2 from the terpy* ligand. Elimination of NO_3 in (b) to yield $[Eu^{II}(terpy^*)(NO_3)]^+$ is also apparent, as are elimination of NO, and other unassigned ligand fragmentations. Comparison of these results with those for lower CID voltages in Supplementary Figures 9 and 10 indicate that the sole terpy* ligand in $[M(terpy^*)(NO_3)_2]^+$ is more strongly bound than the terpy* in $[M(terpy^*)_2(NO_3)_2]^+$. The results here demonstrate control of fragmentation by redox chemistry of the metal center: the sole channel for M = Ce creates Ce(IV), whereas for M = Eu the dominant pathway is ligand dissociation, with creation of Eu(II) a distinctive minor channel.

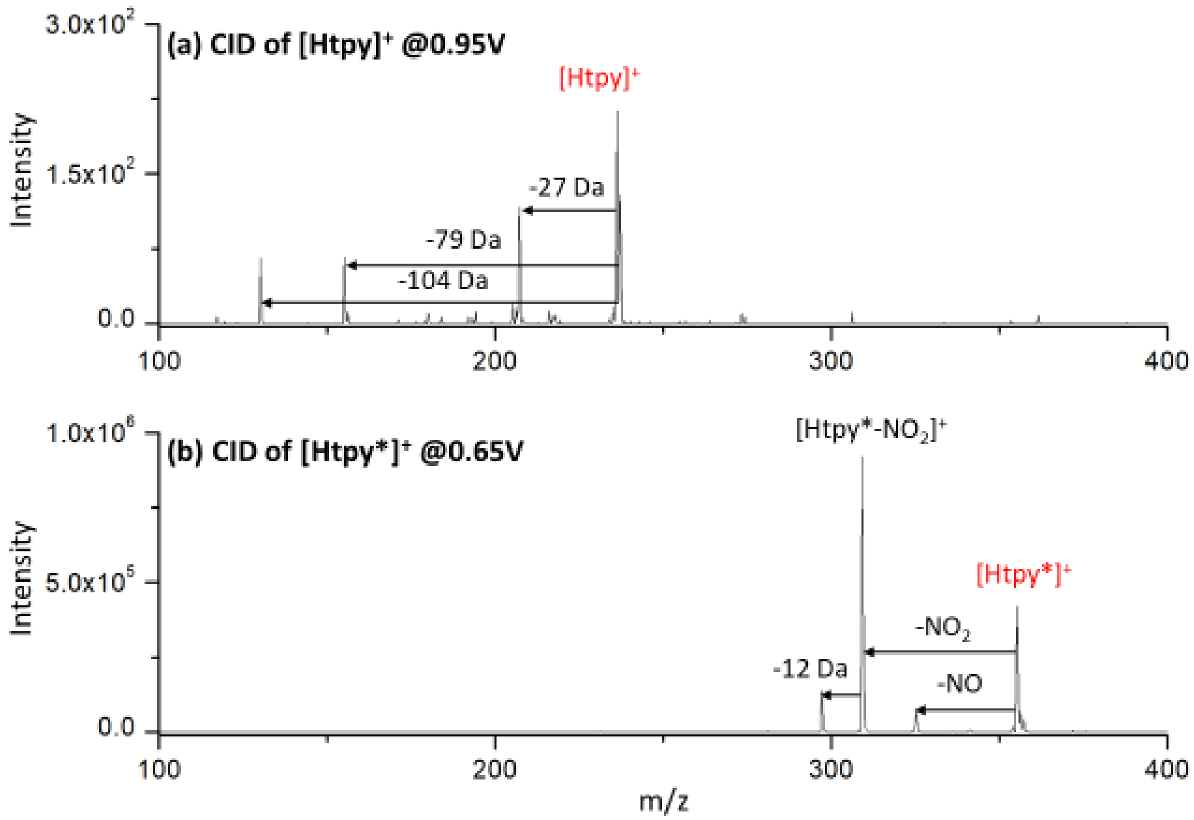

Supplementary Fig. 15 | CID mass spectra of (a) $[H_{terpy}]^+$ at a nominal CID voltage of 0.95 V, and (b) $[H_{terpy*}]^+$ at 0.65 V. The results in (b) show the same fragmentations as are assigned to the terpy* ligand of $[Eu(terpy^*)(NO_3)_2]^+$ in Supplementary Figure 14 (b). The results in (a), where the ligand fragments are not assigned, were obtained at a higher voltage than for (b) and show that $[H_{terpy}]^+$ is more resistant to fragmentation than $[H_{terpy*}]^+$. Elimination of the NO_2 moiety from terpy* is the most facile fragmentation channel for terpy* bound both to a proton in $[H_{terpy*}]^+$ and to the metal center in $[Eu(terpy^*)(NO_3)_2]^+$ (Supplementary Figure 14 (b)). For the $[Ce(terpy^*)(NO_3)_2]^+$ complex, the accessible Ce(IV) oxidation state enables the alternative NO_2-elimination pathway, rather than terpy* ligand decomposition, to yield $[Ce^{IV}O(terpy^*)(NO_3)]^+$ (Supplementary Figure 14 (a)).

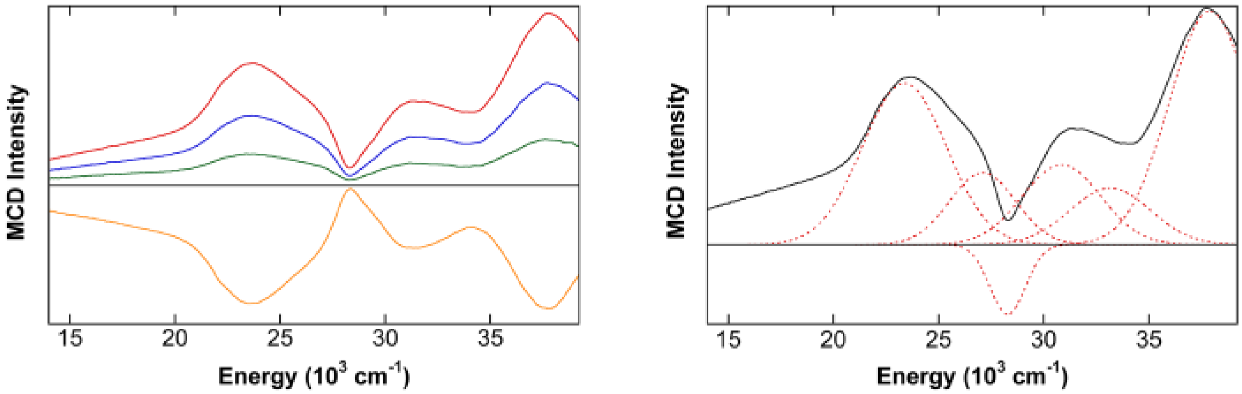

Supplementary Fig. 16 | UV-vis Magnetic Circular Dichroism spectra of Ce1. The UV-vis MCD spectrum of a fluorolube mull of Ce1. Left: The field dependent UV-vis MCD spectrum of **Ce1** (7 T (red), 3.5 T (blue), 1.5 T (green), and -7 T (orange). Right: The 7 T UV-vis MCD spectrum of **Ce1** (black solid). This spectrum can be fit to six transitions (red dashed), as predicted by the computational analysis. Assignments with respect to the calculated transitions can be seen in Supplementary Table 5. Note: tail at low energy is due to scattering.

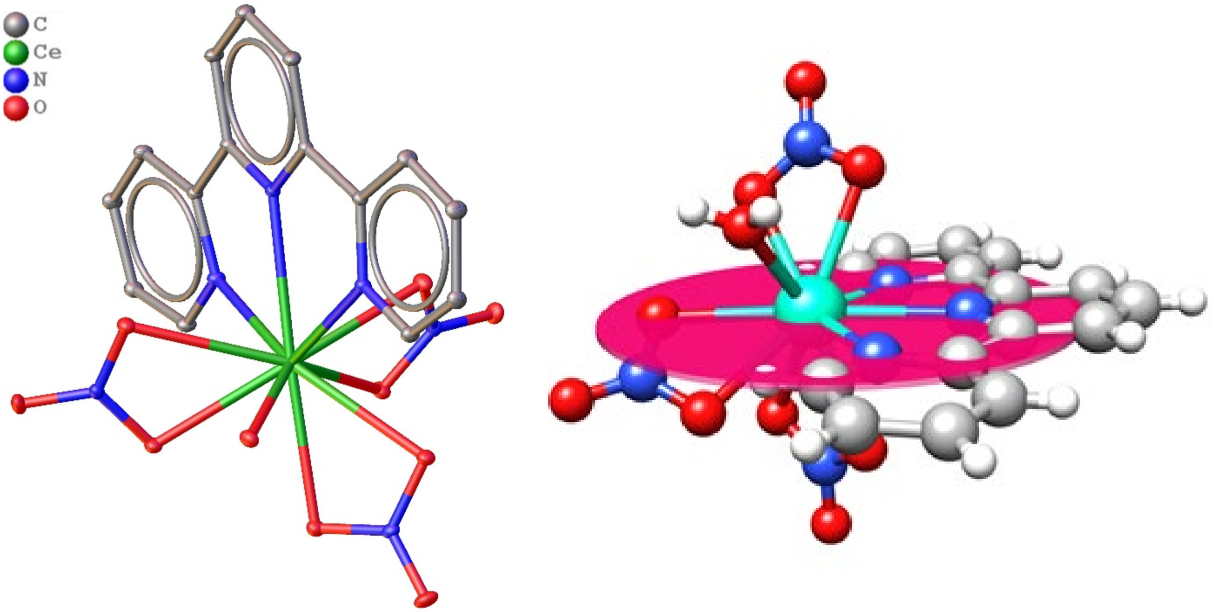

Supplementary Fig. 17 | Ce2 Structure and plane. The structure on the left shows the thermal ellipsoid plot of the **Ce2** crystal structure with hydrogen atoms omitted for clarity, and the plane highlighted on the right including the terpy ligand and nitrate molecule.

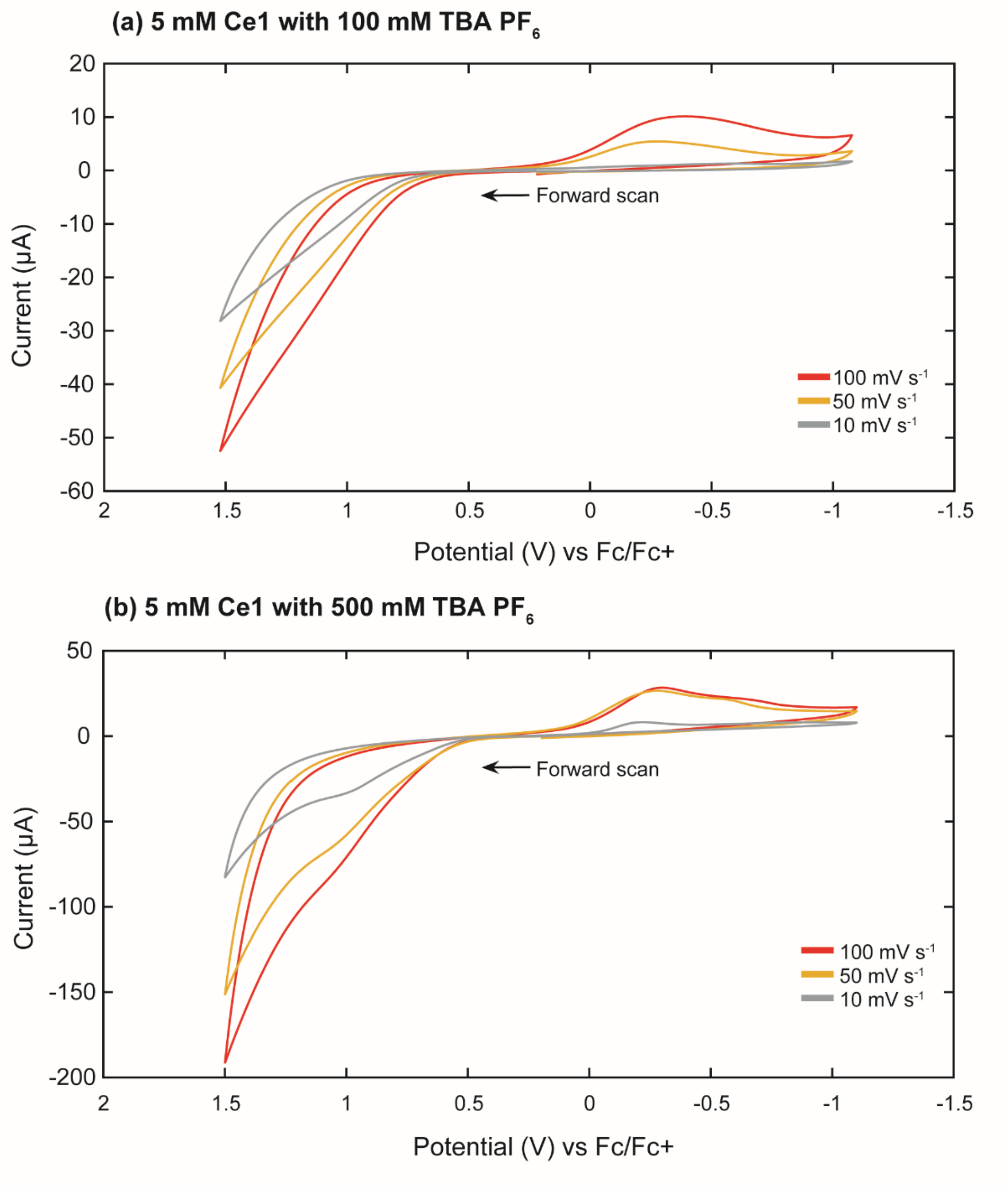

Supplementary Fig. 18 | Cyclic voltammograms of 5 mM Ce1 complex in (a) 100 mM TBA PF_6 and (b) 500 mM TBA PF_6. Data are shown with three different scan rates for each, 10, 50 and 100 mV s^{-1}. Data are corrected to an internal Fc/Fc+ reference, and voltammetry was performed on a 2 mm Pt disk working electrode.

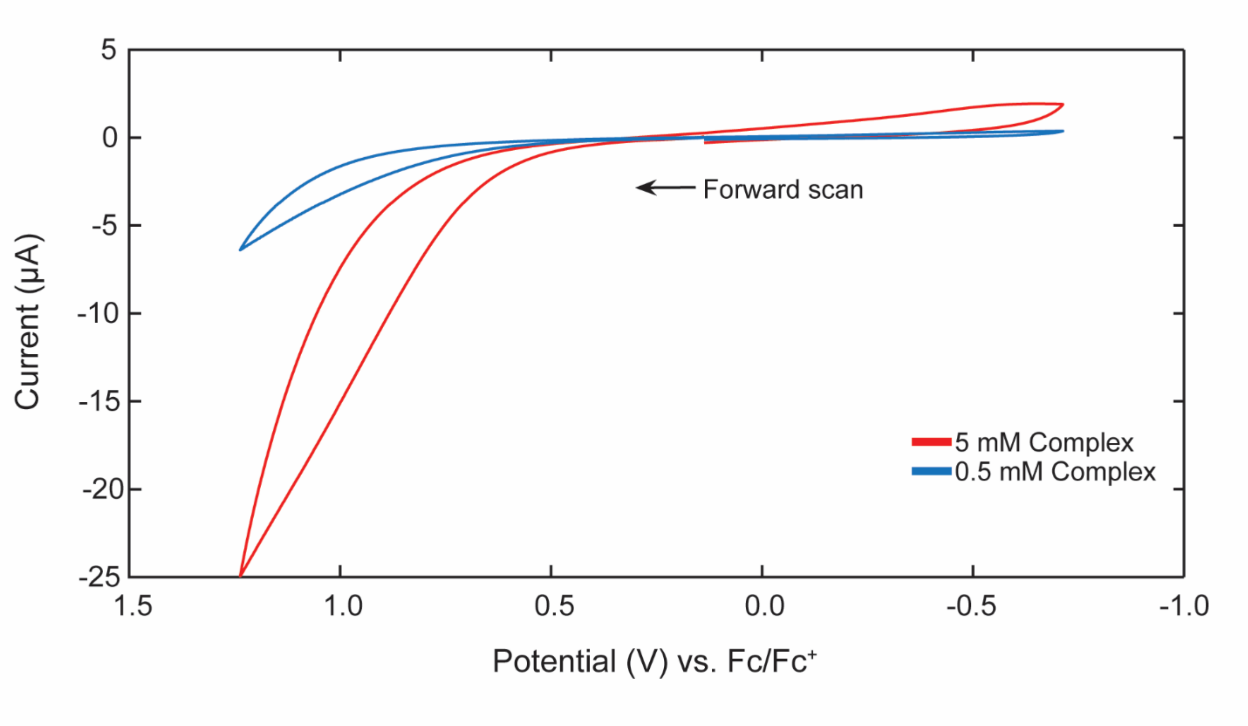

Supplementary Fig. 19 | Cyclic voltammograms of 0.5 mM and 5 mM Ce1 complex. Both solutions were made in 100 mM TBA PF_6 and data are corrected to an internal Fc/Fc^+ reference, with voltammetry performed using a 2 mm Pt disk working electrode.

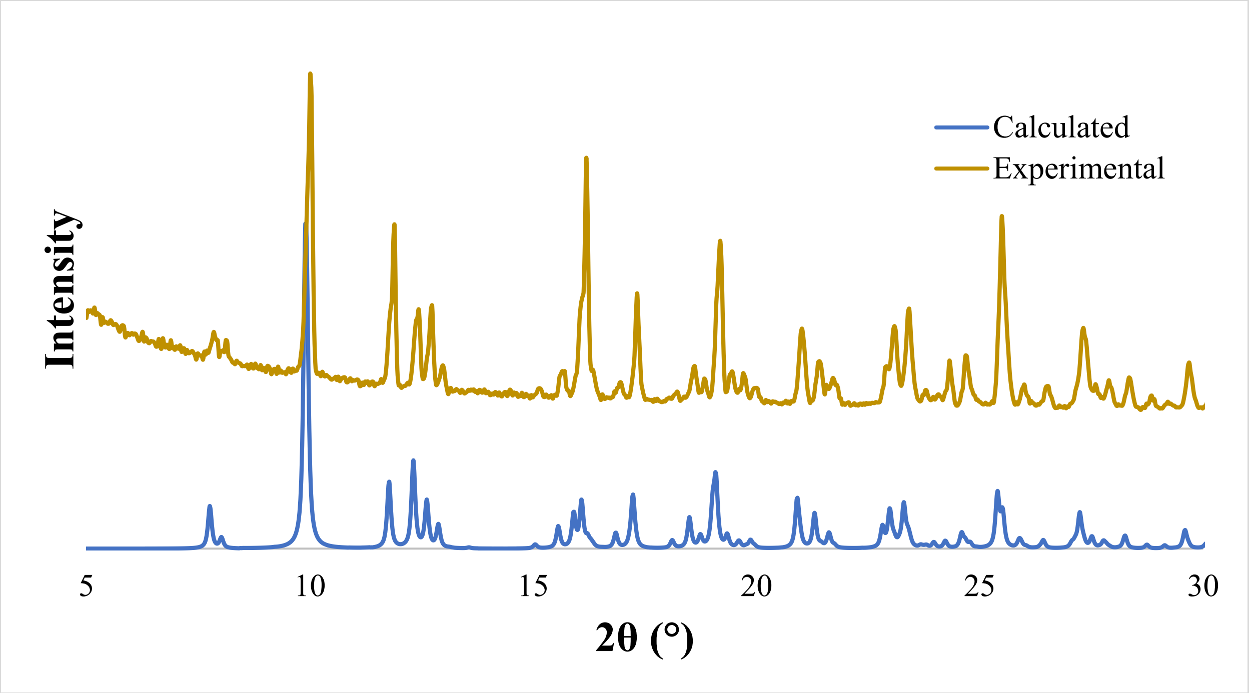

Supplementary Fig. 20 | Experimental and calculated Powder X-ray Diffraction (PXRD) patterns of Ce1.

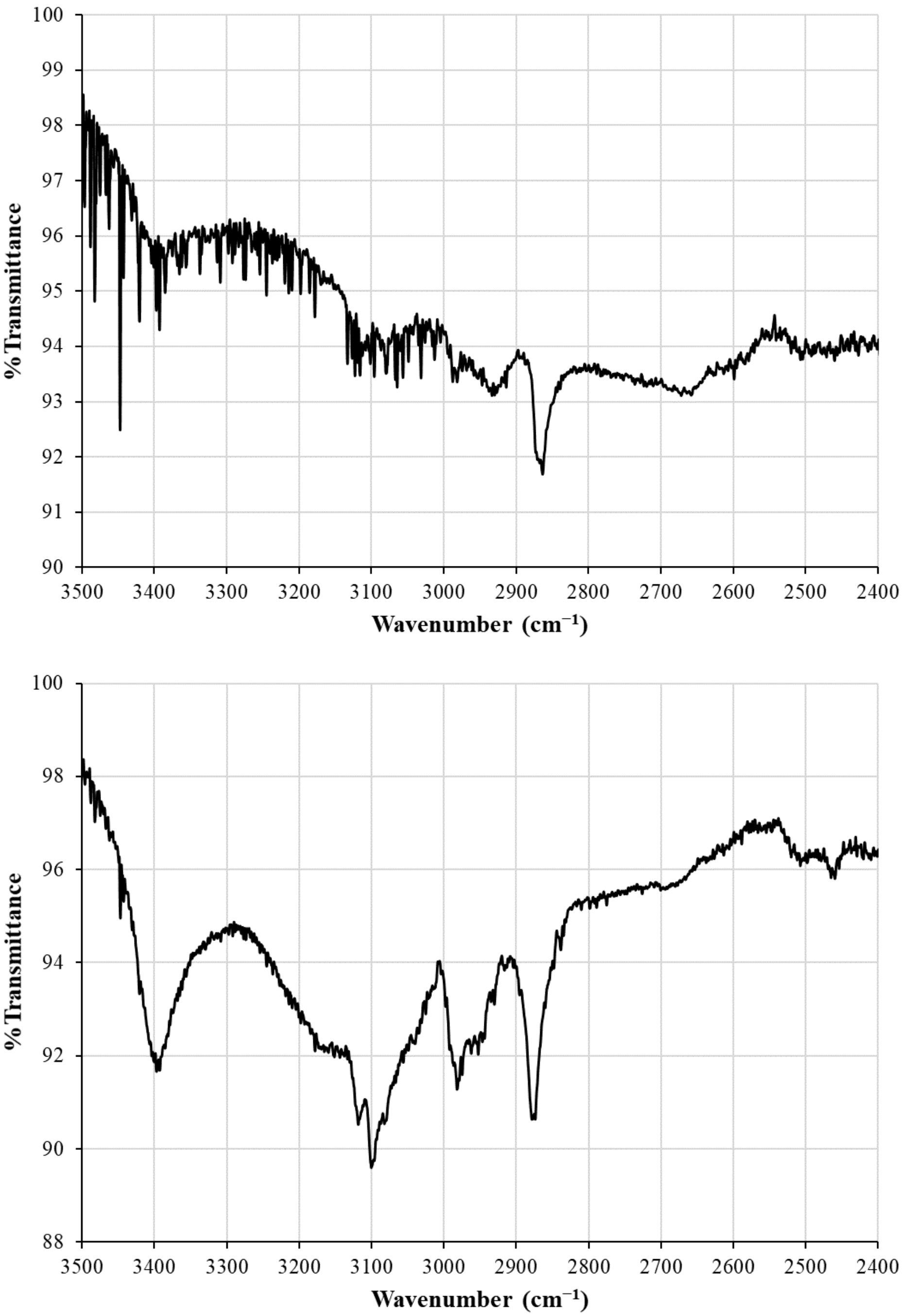

Supplementary Fig. 21 | ATR-IR Spectra of Ce1 (top) and Ce2 (bottom) from 2,400 – 3,500 cm^{-1}.

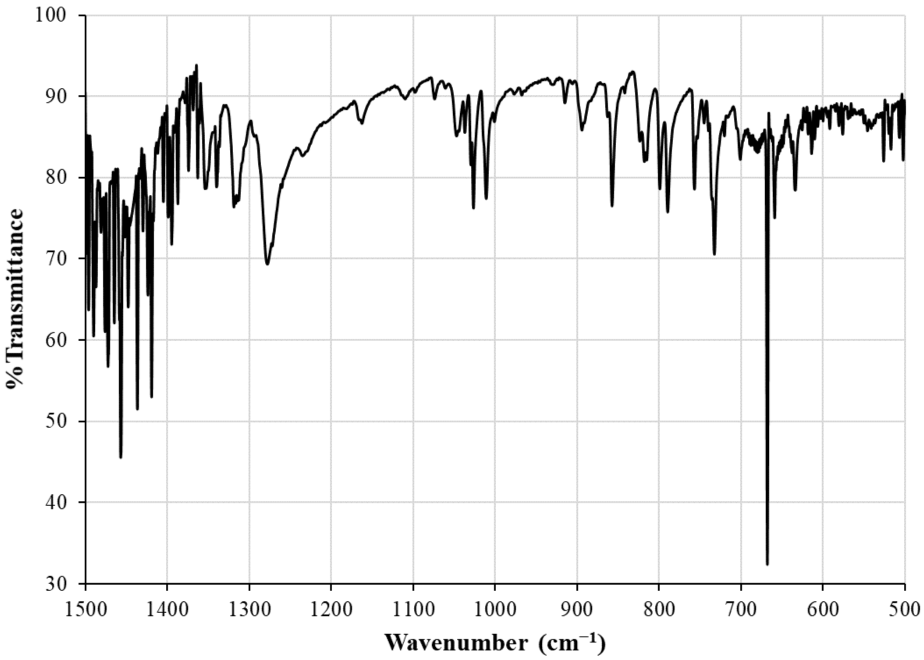

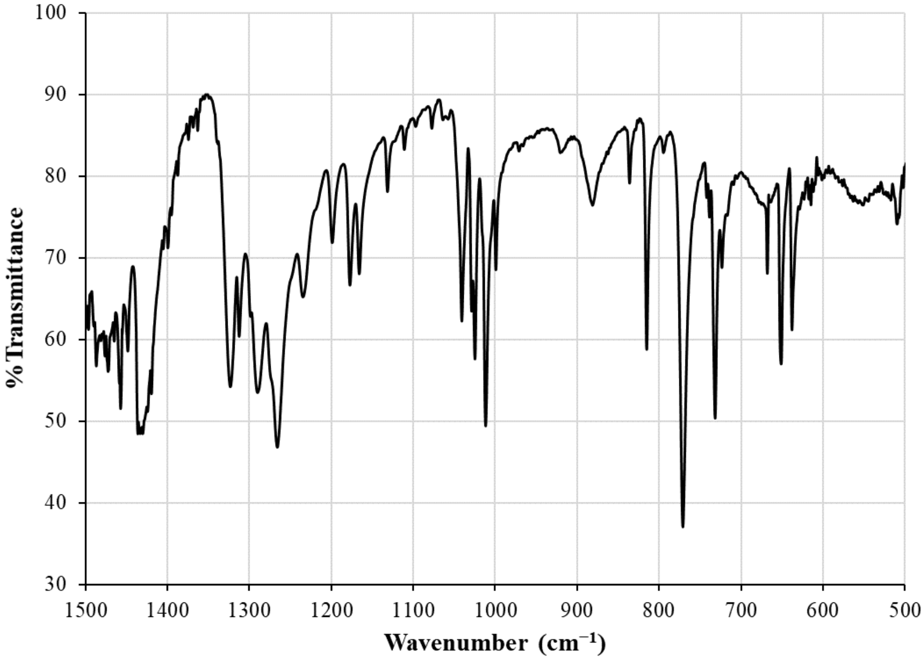

Supplementary Fig. 22 | ATR-IR Spectra of Ce1 (top) and Ce2 (bottom) from 500 – 1,500 cm^{-1}.

4. SUPPLEMENTARY TABLES

Supplementary Table 1 | Spin-orbit states for Bk1. Wavefunctions were obtained using a minimal and extended active spaces and were compared to experimental data reported by Carnall[1] and Varga[2].

SO-State*	CAS+SOC	PT2+SOC
$^7F_6$	0.0	0.0
$^7F_5$	4979.5	5033.7
$^7F_4$	7123.5	6890.2
$^7F_3$	9162.1	9023.5
$^7F_2$	10086.3	9802.9
$^7F_1$	10780.3	10554.7
$^7F_0$	10996.5	10704.8
$^5D_4$	20296.0	18516.8
$^5D_3$	23259.4	24752.9
$^5L_{10}$	27462.6	25569.4

Supplementary Table 2 | Molecular electronic states derived from the $^7F_6$ in Bk1. Wavefunctions were obtained using a minimal SOC-CAS(8,7) and extended SOC-CAS(8,12) active spaces.

**Free Ion Term**	**Molecular State***	**SOC-CAS (8,7)**	**SOC-CAS (8,12)**
	E	0.00	0.00
		9.34	0.14
	A	48.8	77.4 (E)
	A	86.7	79.1
	A	141.6	192.6 (E)
	A	233.7	199.4
$^7F_6$	A	254.6	315.1
	A	279.6	334.8
	A	305.5	391.4
	A	339.9	562.5
	A	349.1	609.7
	A	413.4	623.2
	A	428.5	650.4

Supplementary Table 3 | Composition of the three Kramer's doublets of Ce1. Percentage of contribution of the terms |J,M_J> to the three Kramers doublets derived from the ground multiplet J=5/2 of Ce1.

Component	KD1	KD2	KD3
$\|\frac{5}{2},\mp\frac{5}{2}>$	0.2	1.7	0.0
$\|\frac{5}{2},\mp\frac{3}{2}>$	6.9	19.1	14.0
$\|\frac{5}{2},\mp\frac{1}{2}>$	6.2	8.3	26.0
$\|\frac{5}{2},\pm\frac{1}{2}>$	13.1	38.2	8.2
$\|\frac{5}{2},\pm\frac{3}{2}>$	9.8	3.6	46.5
$\|\frac{5}{2},\pm\frac{5}{2}>$	63.8	29.1	5.3

Supplementary Table 4 | Ab-initio calculated crystal-field parameters B(k,q), where *k* corresponds to the rank and *q* the projection of the operator.

**k**	**Q**	**B(k,q)**
2	-2	-10.7
2	-1	12.1
2	0	-6.93
2	1	11.2
2	2	9.25
4	-4	1.27
4	-3	2.39
4	-2	1.99
4	-1	0.24
4	0	-0.30
4	1	1.55
4	2	4.76
4	3	2.50
4	4	-0.15

Supplementary Table 5 | TD-DFT and Magnetic Circular Dichroism transitions. Assignment of MCD transitions based on a BP86/STO-TZP TD-DFT calculation on **Ce1**. The f_{osc} corresponds to the oscillator strength that is related to the absorbance of the sample. MCD transition refers to the experimentally observed values from Supplementary Fig. 16.

**Wavelength (nm)**	**TD-DFT (cm^{-1})**	**MCD Transition (cm^{-1})**	**f_{osc}**	**Assignment**
431	23117	23450	0.0086	4*f* → terpy*
428	27068	27500	0.0020	NO_3^- → 4*f*
403	28477	28250	0.0020	Lig → 4*f*
318	29800	30700	0.0141	Lig → 4*f*
276	33640	33450	0.0005	Lig → 4*f*
275	-	37900	-	Lig → 4*f*

Supplementary Table 6 | QTAIM metrics at the bond critical point (BCP) for Ce1. The analysis is based on scalar relativistic CAS(8,7) densities. The electron density ρ(r) is given in |e| $Å^{-3}$; and potential V(r), kinetic G(r), and total H(r) energy densities are given in kJ mol^{-1} $Å^{-3}$.

Bond	$\rho_{BCP}(r)$	$\delta_{BCP}(r)$	|V(r)|	G(r)	|V(r)|/G(r)	$H_{BCP}(r)$	ε
Ce-Nmid	0.2733	0.1727	-628.2	615.9	1.02	-12.3	0.0425
Ce-Nter	0.2733	0.1891	-659.7	635.2	1.04	-24.6	0.0407
Ce-Nter	0.2612	0.1878	-661.5	638.7	1.04	-22.8	0.0467
Ce-OH2	0.3003	0.1862	-870.3	868.5	1.00	-1.8	0.1045
Ce-O1	0.2531	0.163	-665.0	673.8	0.99	8.8	0.0639
Ce-O2	0.2436	0.1554	-635.2	651.0	0.98	15.8	0.0592
Ce-O1	0.2645	0.1694	-710.6	712.4	1.00	1.8	0.0632
Ce-O2	0.2429	0.1508	-635.2	652.7	0.97	17.5	0.0532
Ce-O1	0.2490	0.1728	-731.7	728.2	1.00	-3.5	0.0698
Ce-O2	0.2713	0.1542	-656.2	670.3	0.98	14.0	0.0483

Supplementary Table 7 | QTAIM metrics at the bond critical point (BCP) for Bk1. The analysis is based on scalar relativistic CAS(8,7) densities. The electron density ρ(r) is given in |e| $Å^{-3}$; and potential V(r), kinetic G(r), and total H(r) energy densities are given in kJ mol^{-1} $Å^{-3}$.

Bkterpy	$\rho_{BCP}(r)$	$\delta_{BCP}(r)$	V(r)	G(r)	|V(r)|/G(r)	$H_{BCP}(r)$	ε
Bk-Nmid	0.3199	0.2058	-905.4	868.5	1.0424	-36.8	0.0344
Bk-Nter	0.3145	0.2121	-877.3	838.7	1.0460	-38.6	0.0425
Bk-Nter	0.3104	0.2073	-859.8	822.9	1.0448	-36.8	0.0278
Bk-OH2	0.3550	0.2145	-1175.6	1152.8	1.0198	-22.8	0.0650
Bk-O1	0.2848	0.1759	-858.0	856.3	1.0020	-1.8	0.0445
Bk-O2	0.2672	0.1617	-791.3	800.1	0.9890	8.8	0.0278
Bk-O1	0.2996	0.1865	-917.7	907.1	1.0116	-10.5	0.0430
Bk-O2	0.2375	0.1404	-682.5	707.1	0.9653	24.6	0.0275
Bk-O1	0.3023	0.1789	-937.0	931.7	1.0056	-5.3	0.0371
Bk-O2	0.2639	0.1588	-777.3	787.8	0.9866	10.5	0.0245

Supplementary Table 8 | Interacting Quantum Atom (IQA) energy decomposition analysis. IQA decomposes the energy into Coulomb and exchange energies, which are related to classical and quantum mechanical (covalent) interactions, respectively. Energies are given in eV.

Bond	$E_{Coulomb}$		$E_{exchange}$		$E_{interaction}$	
	**Ce1**	**Bk1**	**Ce1**	**Bk1**	**Ce1**	**Bk1**
M - N_{mid}	-19.2	-20.4	-20.7	-21.6	-39.9	-41.9
M – N_{ter}	-19.3	-19.9	-20.8	-21.2	-40.1	-41.1
M – N_{ter}	-20.1	-20.4	-21.1	-21.6	-41.2	-42.0
M – OH_2	-19.4	-18.7	-20.4	-20.0	-39.8	-38.6
M – O1	-10.2	-10.2	-11.1	-11.3	-21.2	-21.5
M – O2	-9.8	-9.8	-10.7	-10.7	-20.6	-20.5
M – O1	-10.5	-10.6	-11.4	-11.7	-21.9	-22.3
M – O2	-9.7	-9.5	-10.6	-10.3	-20.3	-19.8
M – O1	-10.3	-10.4	-11.2	-11.4	-21.5	-21.8
M – O2	-10.2	-10.3	-11.1	-11.2	-21.3	-21.6
Total M - L	-138.6	-140.2	-149.2	-150.9	-287.8	-291.2

Supplementary Table 9 | Crystal data and structure refinement for Ce1

Empirical formula	$C_{25}H_{24}CeN_7O_{13}$
Formula weight (g mol^{-1})	770.63
Temperature/K	28
Crystal system	monoclinic
Space group	$P2_1/c$
a/Å	11.5035(19)
b/Å	17.685(3)
c/Å	14.442(2)
α/°	90
β/°	105.210(4)
γ/°	90
Volume/Å^3	2835.1(8)
Z	4
ρ_{calc} g/cm^3	1.805
μ/mm^{-1}	1.688
F(000)	1540.0
Crystal size/mm^3	0.198 × 0.093 × 0.066
Radiation	MoK_α (λ = 0.71073 Å)
2Θ range for data collection/°	4.332 to 66.462
Index ranges	-17 ≤ h ≤ 17, -27 ≤ k ≤ 27, -22 ≤ l ≤ 22
Reflections collected	85127
Independent reflections	10850 [R_{int} = 0.1351, R_{sigma} = 0.0829]
Data/restraints/parameters	10850/0/420
Goodness-of-fit on F^2	1.053
Final R indexes [I≥2σ (I)]	R_1 = 0.0450, wR_2 = 0.0721
Final R indexes [all data]	R_1 = 0.0837, wR_2 = 0.0804
Largest diff. peak/hole / e Å^{-3}	0.87/-0.85

Supplementary Table 10 | Bond Lengths for Ce1

Atom	Atom	Length/Å	Atom	Atom	Length/Å
Ce01	O003	2.6111(19)	N00K	C00O	1.344(3)
Ce01	O004	2.6123(19)	N00K	C00X	1.353(3)
Ce01	O006	2.5992(19)	N00L	C00R	1.356(3)
Ce01	O009	2.491(2)	N00L	C015	1.344(3)
Ce01	O00B	2.6013(19)	C00M	C00R	1.493(4)
Ce01	O00D	2.567(2)	C00M	C014	1.393(4)
Ce01	O00E	2.580(2)	C00N	C00R	1.393(4)
Ce01	N00F	3.019(2)	C00N	C013	1.390(4)
Ce01	N00H	2.628(2)	C00O	C00S	1.387(4)
Ce01	N00J	3.012(2)	C00P	C00Q	1.385(4)
Ce01	N00K	2.629(2)	C00P	C00T	1.395(4)
Ce01	N00L	2.640(2)	C00Q	C014	1.386(4)
O002	N00F	1.233(3)	C00S	C012	1.380(4)
O003	N00G	1.279(3)	C00U	C018	1.537(4)
O004	N00F	1.269(3)	C00V	C017	1.513(4)
O005	C00U	1.446(3)	C00V	C018	1.536(4)
O005	C017	1.439(3)	C00W	C00Z	1.390(4)
O006	N00F	1.276(3)	C00W	C016	1.404(4)
O007	N00I	1.234(3)	C00X	C00Y	1.397(4)
O008	N00J	1.219(3)	C00X	C015	1.494(4)
O00A	N00G	1.223(3)	C00Y	C012	1.393(4)
O00B	N00J	1.277(3)	C00Z	C011	1.385(4)
O00C	N00I	1.232(3)	C010	C013	1.394(4)
O00D	N00J	1.284(3)	C010	C015	1.400(4)
O00E	N00G	1.282(3)	C011	C01A	1.390(4)
N00H	C00M	1.358(3)	C013	C016	1.485(4)
N00H	C00T	1.345(3)	C016	C019	1.401(4)
N00I	C011	1.467(3)	C019	C01A	1.387(4)

Supplementary Table 11 | Crystal data and structure refinement for Bk1

Empirical formula	$C_{25}H_{24}BkN_7O_{13}$
Formula weight (g mol^{-1})	877.51
Temperature/K	28
Crystal system	monoclinic
Space group	$P2_1/c$
a/Å	11.5369(18)
b/Å	17.590(3)
c/Å	14.325(2)
α/°	90
β/°	106.110(4)
γ/°	90
Volume/Å^3	2793.0(7)
Z	4
ρ_{calc}g/cm^3	2.087
μ/mm^{-1}	3.002
F(000)	1696.0
Crystal size/mm^3	0.215 × 0.128 × 0.085
Radiation	MoKα (λ = 0.71073 Å)
2Θ range for data collection/°	4.344 to 56.782
Index ranges	-15 ≤ h ≤ 15, -23 ≤ k ≤ 23, -19 ≤ l ≤ 19
Reflections collected	87020
Independent reflections	6995 [R_{int} = 0.0766, R_{sigma} = 0.0325]
Data/restraints/parameters	6995/0/420
Goodness-of-fit on F^2	1.087
Final R indexes [I≥2σ (I)]	R_1 = 0.0240, wR_2 = 0.0504
Final R indexes [all data]	R_1 = 0.0374, wR_2 = 0.0542
Largest diff. peak/hole / e Å^{-3}	0.78/-1.42

Supplementary Table 12 | Bond Lengths for Bk1

Atom	Atom	Length/Å	Atom	Atom	Length/Å
Bk1	O3	2.550(3)	C18	C32	1.385(5)
Bk1	N4	2.551(3)	N19	O20	1.235(4)
Bk1	O7	2.494(3)	N19	C34	1.473(4)
Bk1	O8	2.527(3)	C22	C29	1.392(5)
Bk1	N9	2.544(3)	C22	C45	1.387(5)
Bk1	N13	2.530(3)	C23	C25	1.390(5)
Bk1	O14	2.556(3)	O26	N49	1.244(4)
Bk1	N41	2.950(3)	C29	C33	1.492(5)
Bk1	N49	2.927(3)	C30	C32	1.387(5)
Bk1	O1AA	2.506(3)	C30	C33	1.394(5)
Bk1	O2	2.594(3)	C32	C43	1.490(5)
Bk1	O0AA	2.410(3)	C34	C40	1.376(5)
O3	N41	1.260(4)	C34	C46	1.383(5)
N4	C29	1.345(4)	C35	C37	1.394(5)
N4	C7	1.336(4)	C36	C43	1.391(5)
O5	N41	1.237(4)	C36	C46	1.390(5)
O6	N19	1.223(4)	C40	C13	1.385(5)
O7	N49	1.220(4)	C43	C13	1.404(5)
O8	N41	1.278(4)	C45	C9	1.376(5)
N9	C23	1.357(4)	O1AA	N6	1.290(4)
N9	C35	1.344(4)	O2	N6	1.265(4)
C11	C25	1.382(5)	N6	O2AA	1.224(4)
C11	C37	1.384(5)	C7	C9	1.387(5)
N13	C16	1.349(4)	C8	O1	1.443(4)
N13	C33	1.342(4)	C8	C3	1.526(5)
O14	N49	1.284(4)	C10	C0AA	1.507(5)
C16	C18	1.392(5)	C10	C3	1.527(5)
C16	C23	1.484(5)	C0AA	O1	1.440(5)

Supplementary Table 13 | Crystal data and structure refinement for Ce2

Empirical formula (g mol^{-1})	$C_{19}H_{21}CeN_6O_{11}$
Formula weight	649.54
Temperature/K	28
Crystal system	monoclinic
Space group	$P2_1/c$
a/Å	9.7775(11)
b/Å	15.6553(17)
c/Å	15.2718(17)
α/°	90
β/°	97.237(4)
γ/°	90
Volume/Å^3	2319.0(4)
Z	4
ρ_{calc}g/cm^3	1.860
μ/mm^{-1}	2.036
F(000)	1292.0
Crystal size/mm^3	0.34 × 0.218 × 0.09
Radiation	MoK_α (λ = 0.71073 Å)
2Θ range for data collection/°	4.94 to 96.854
Index ranges	-20 ≤ h ≤ 20, -32 ≤ k ≤ 32, -32 ≤ l ≤ 31
Reflections collected	421077
Independent reflections	22448 [R_{int} = 0.0525, R_{sigma} = 0.0193]
Data/restraints/parameters	22448/0/342
Goodness-of-fit on F^2	1.076
Final R indexes [I≥2σ (I)]	R_1 = 0.0222, wR_2 = 0.0448
Final R indexes [all data]	R_1 = 0.0286, wR_2 = 0.0464
Largest diff. peak/hole / e Å^{-3}	1.11/-2.31

Supplementary Table 15 | Bond Lengths for Ce2

Atom	Atom	Length/Å	Atom	Atom	Length/Å
Ce1	O2	2.5891(6)	N11	C18	1.3504(8)
Ce1	O3	2.5669(6)	N11	C29	1.3417(8)
Ce1	N4	3.0045(6)	N13	O21	1.2346(8)
Ce1	N5	2.6646(6)	N14	O17	1.2217(8)
Ce1	O6	2.6283(6)	C16	C19	1.4861(9)
Ce1	N7	2.6106(6)	C16	C26	1.3967(9)
Ce1	O8	2.5814(6)	C18	C23	1.4848(8)
Ce1	O9	2.6588(6)	C18	C32	1.3986(9)
Ce1	N11	2.6266(6)	C19	C24	1.3957(9)
Ce1	N14	3.0101(6)	C22	C29	1.3896(10)
Ce1	O15	2.5224(6)	C22	C31	1.3885(10)
Ce1	O1	2.5267(6)	C23	C30	1.3966(9)
O2	N14	1.2759(8)	C24	C25	1.3888(10)
O3	N13	1.2665(8)	C25	C30	1.3859(9)
N4	O6	1.2717(8)	C26	C33	1.3910(10)
N4	O15	1.2809(8)	C28	C38	1.3865(9)
N4	O20	1.2207(8)	C31	C32	1.3902(9)
N5	C19	1.3519(8)	C33	C38	1.3864(10)
N5	C23	1.3524(8)	O0AA	C2	1.4545(9)
N7	C16	1.3487(8)	O0AA	C1	1.4447(9)
N7	C28	1.3428(9)	C2	C3	1.5253(10)
O8	N14	1.2765(8)	C1	C0AA	1.5144(10)
O9	N13	1.2655(8)	C0AA	C3	1.5311(12)

5. SUPPLEMENTARY REFERENCES

1. Carnall, W., Beitz, J. & Crosswhite, H. Electronic energy level and intensity correlations in the spectra of the trivalent actinide aquo ions. III. Bk3+. *J. Chem. Phys.* **80**, 2301–2308 (1984).

2. Ma, C. G. *et al.* Energy level schemes of fN electronic configurations for the di-, tri-, and tetravalent lanthanides and actinides in a free state. *J. Lumin.* **170**, 369–374 (2016).

3. Silver, M. A. *et al.* Electronic Structure and Properties of Berkelium Iodates. *J. Am. Chem. Soc.* **139**, 13361–13375 (2017).

4. Cary, S. K. *et al.* Emergence of californium as the second transitional element in the actinide series. *Nat. Commun.* **6**, 6827 (2015).

5. Sperling, J. M. *et al.* Compression of curium pyrrolidine-dithiocarbamate enhances covalency. *Nature* **583**, 396–399 (2020).

6. Albrecht-Schmitt, T. E., Hobart, D. E., Páez-Hernández, D. & Celis-Barros, C. Theoretical examination of covalency in berkelium(IV) carbonate complexes. *Int. J. Quantum Chem.* **120**, (2020).

7. Frenking, G. & Fröhlich, N. The Nature of the Bonding in Transition-Metal Compounds. *Chem. Rev.* **100**, 717–774 (2000).

8. Lucena, A. F. *et al.* Synthesis and hydrolysis of gas-phase lanthanide and actinide oxide nitrate complexes: a correspondence to trivalent metal ion redox potentials and ionization energies. *Phys. Chem. Chem. Phys.* **17**, 9942–9950 (2015).

9. Heinemann, C., Cornehl, H. H., Schröder, D., Dolg, M. & Schwarz, H. The CeO2+ Cation: Gas-Phase Reactivity and Electronic Structure. *Inorg. Chem.* **35**, 2463–2475 (1996).

10. Rios, D. *et al.* Gas-Phase Uranyl, Neptunyl, and Plutonyl: Hydration and Oxidation Studied by Experiment and Theory. *Inorg. Chem.* **51**, 6603–6614 (2012).

11. Rios, D. *et al.* Electron transfer dissociation of dipositive uranyl and plutonyl coordination complexes. *J. Mass Spectrom.* **46**, 1247–1254 (2011).

12. Aquilante, F. *et al.* MOLCAS 7: The Next Generation. *J. Comput. Chem.* **31**,

224–247 (2010).

13. Wolf, A., Reiher, M. & Hess, B. A. The generalized Douglas–Kroll transformation. *J. Chem. Phys.* **117**, 9215–9226 (2002).

14. Roos, B. O., Taylor, P. R. & Siegbahn, P. E. M. A complete active space SCF method (CASSCF) using a density matrix formulated super-CI approach. *Chem. Phys.* **48**, 157–173 (1980).

15. Andersson, K., Malmqvist, P. A., Roos, B. O., Sadlej, A. J. & Wolinski, K. Second-order perturbation theory with a CASSCF reference function. *J. Phys. Chem.* **94**, 5483–5488 (1990).

16. Malmqvist, P. Å., Roos, B. O. & Schimmelpfennig, B. The restricted active space (RAS) state interaction approach with spin–orbit coupling. *Chem. Phys. Lett.* **357**, 230–240 (2002).

17. Heß, B. A., Marian, C. M., Wahlgren, U. & Gropen, O. A mean-field spin-orbit method applicable to correlated wavefunctions. *Chem. Phys. Lett.* **251**, 365–371 (1996).

18. Ungur, L. & Chibotaru, L. F. SINGLE_ANISO program in MOLCAS.

19. Stevens, K. W. H. Matrix Elements and Operator Equivalents Connected with the Magnetic Properties of Rare Earth Ions. *Proc. Phys. Soc. Sect. A* **65**, 209–215 (1952).

20. Bickelhaupt, F. M. & Baerends, E. J. Kohn-Sham Density Functional Theory: Predicting and Understanding Chemistry. in 1–86 (2007).

21. AIMAll (Version 19.10.12), Todd A. Keith, TK Gristmill Software, Overland Park KS, USA, 2019, aim.tkgristmill.com.

22. te Velde, G. *et al.* Chemistry with ADF. *J. Comput. Chem.* **22**, 931–967 (2001).

23. ADF2019.3, SCM, Theoretical Chemistry, Vrije Universiteit, Amsterdam, The Netherlands, http://www.scm.com.

24. Ramanantoanina, H., Urland, W., Cimpoesu, F. & Daul, C. Ligand field density functional theory calculation of the $4f^2 \rightarrow 4f^1 5d^1$ transitions in the quantum cutter $Cs_2KYF_6{:}Pr^{3+}$. *Phys. Chem. Chem. Phys.* **15**, 13902–13910 (2013).

25. Silver, M. A. *et al.* Characterization of berkelium(III) dipicolinate and borate compounds in solution and the solid state. *Science.* **353**, aaf3762–aaf3762 (2016).
