# Supplementary material for: Creation of an unexpected plane of enhanced covalency in cerium(III) and berkelium(III) terpyridyl complexes
Source: Nat Commun. 2021 Dec 10;12:7230. doi: 10.1038/s41467-021-27576-y (PMC8664847; doi:10.1038/s41467-021-27576-y)
Supplement: Supplementary file 3 — Description of Additional Supplementary Files [file 41467_2021_27576_MOESM3_ESM.pdf]

Description of Additional Supplementary Files

Supplementary Data 1: Atomic coordinates used for electronic structure calculations
