# Supplementary material for: Creation of an unexpected plane of enhanced covalency in cerium(III) and berkelium(III) terpyridyl complexes
Source: Nat Commun. 2021 Dec 10;12:7230. doi: 10.1038/s41467-021-27576-y (PMC8664847; doi:10.1038/s41467-021-27576-y)
Supplement: Supplementary file 4 — Supplementary Data 1 [file 41467_2021_27576_MOESM4_ESM.pdf]

Coordinates of Ce1

Ce	0.000000000	0.000000000	0.000000000
N	0.000000000	0.000000000	3.030333437
N	2.560722877	0.000000000	0.642516813
N	0.566414240	-0.513583904	-2.913832028
N	1.272177498	2.293522842	-0.165600924
N	-2.504353207	1.609466250	-0.500814867
N	9.629509574	0.499874445	5.341719758
N	1.238417863	-2.310060039	0.202347797
O	-2.001054006	-1.481514043	-0.077088700
O	-3.619881290	2.078840691	-0.734485792
O	0.297729509	1.054338911	2.370061542
O	-2.101248061	1.394011352	0.682718964
O	-1.707089901	1.315435944	-1.453086017
O	10.659851006	0.967678812	4.849616259
O	0.696047452	-0.564985414	-4.124954426
H	-2.393534723	-1.473477704	0.683050346
O	0.051626654	-0.036415084	4.250990825
O	-0.343737819	-1.165446019	-2.300080724
O	9.542428701	0.110583202	6.507804726
O	1.343274335	0.196464531	-2.178827817
O	-0.350828360	-1.023598020	2.341784606
C	2.555589096	2.401000593	0.265464995
C	4.357581808	1.276358993	1.605570368
H	4.740701199	2.125904371	1.791986193
C	0.557177674	-3.436359281	-0.064676879
H	-0.319269139	-3.364079178	-0.425242435
C	1.433140395	4.535941307	-1.036535319
H	1.023144684	5.256335522	-1.501027491
C	2.736595285	4.639255603	-0.579478261
H	3.232811088	5.438363669	-0.712601228
C	3.173533865	1.186847518	0.878000567
C	1.078855677	-4.701545845	0.163339141
H	0.562477881	-5.478597242	-0.016211038
C	0.735907750	3.351362948	-0.799703623
H	-0.162957573	3.289339612	-1.100672930
C	7.212070952	1.111254464	2.578629013
H	7.115600659	1.682535013	1.825729942
C	2.484970418	-2.409590209	0.718196060
C	3.086170442	-3.651733379	0.936640868
H	3.973666714	-3.705940100	1.271385748
C	8.351871745	1.192475562	3.370187079
H	9.058850650	1.784978431	3.143142882
C	4.393175624	-1.107656238	1.733780527
H	4.817404224	-1.921228359	1.980168020
C	8.435319399	0.391338403	4.496440998
C	2.363544994	-4.807857263	0.656609502
H	2.751055953	-5.662996405	0.803234209
C	4.975047571	0.116714858	2.057980524
C	3.306640067	3.558045886	0.075250328
H	4.201344294	3.605989419	0.390989199
C	3.176857733	-1.125614382	1.041078623
C	6.202890472	0.185772536	2.890516891

C	6.339043916	-0.643101988	4.011911111
H	5.672491364	-1.291861098	4.204631316
C	7.442685662	-0.519084992	4.842088015
H	7.519147864	-1.044686125	5.629855823
H	-2.433144370	-1.635583454	-0.766745993

Coordinates of Bk1

Bk	0.000000000	0.000000000	0.000000000
O	-2.050072970	-1.340260549	-0.710150730
O	-3.558907011	-2.073647531	0.695983157
O	9.354842071	0.064748906	-6.608796220
O	1.339893411	-0.187688401	2.095240249
O	-1.664729913	-1.265069769	1.419050876
O	-0.349108751	1.115685720	2.273206293
O	10.462821319	-0.893156378	-5.010515788
O	0.759151935	0.521904205	4.058449281
O	-0.315224569	1.024689750	-2.264909630
O	0.265772479	-1.063679307	-2.351266891
O	-1.946856842	1.417885400	0.086722801
O	0.051781269	0.075026745	-4.202518332
N	1.133323219	2.279728641	-0.152950916
N	1.161962821	-2.258514063	0.142262705
N	2.434920712	0.000000000	-0.686264314
N	9.434372268	-0.391203902	-5.476144220
N	-2.452509463	-1.572909034	0.461226916
N	0.610727818	0.464193478	2.824561673
N	0.000000000	0.000000000	-2.982692964
H	3.047209212	-5.471074803	0.492934846
H	4.601482116	-2.094980698	-1.887617031
H	3.809388268	3.712736102	-1.298687527
H	4.006088288	-3.644418033	-0.612915183
H	4.669770180	1.942769202	-2.007326545
H	-0.252600431	-3.236452442	1.121922831
H	6.930903199	-1.701345237	-2.006234853
H	0.880262091	-5.258065640	1.385438072
H	7.366135601	1.200737172	-5.665023482
H	2.596205857	5.650046274	-0.751416861
H	8.857594239	-1.787213305	-3.350704816
H	-2.358998462	1.382752148	-0.661784363
H	-0.401902003	3.304325139	0.548194842
H	0.445157031	5.433341073	0.147569336
H	5.540652715	1.411277194	-4.207561264
H	-2.423526039	1.561360053	1.073902287
C	2.567743223	-4.656094118	0.405880945
C	3.040062165	-1.177321137	-0.945237395
C	4.216986636	-1.250086121	-1.686444987
C	2.936108200	3.647180386	-0.928884463
C	2.418560811	-2.388668499	-0.353690341
C	3.133183025	-3.575239585	-0.243751334
C	2.355014551	2.403753432	-0.702963614
C	4.246544070	1.125130131	-1.772975887
C	4.823229492	-0.085659239	-2.126180618
C	3.043002194	1.131812005	-1.071534626

C	8.246453086	-0.301480603	-4.611443184
C	0.626210147	-3.318410700	0.769432605
C	7.032055711	-1.093378359	-2.729431146
C	1.291113407	-4.532757829	0.929913845
C	7.285109335	0.638644665	-4.904445896
C	6.047540410	-0.141015704	-2.972633855
C	2.220461181	4.789553239	-0.604167401
C	8.163833965	-1.165110785	-3.534148797
C	0.459135364	3.390877608	0.157266234
C	0.960832084	4.664163325	-0.065676093
C	6.194781366	0.739367807	-4.055956417
